# Nanoparticles for Cancer Immunotherapy: Innovations and Challenges

**DOI:** 10.3390/ph18081086

**Published:** 2025-07-22

**Authors:** Mohannad M. Fallatah, Ibrahim Alradwan, Nojoud Alfayez, Alhassan H. Aodah, Mohammad Alkhrayef, Majed Majrashi, Yahya F. Jamous

**Affiliations:** 1 Advanced Diagnostics and Therapeutics Institute, Health Sector, King Abdulaziz City for Science and Technology (KACST), Riyadh 11442, Saudi Arabia; ialradwan@kacst.gov.sa (I.A.); nalfayez@kacst.gov.sa (N.A.); aaodah@kacst.gov.sa (A.H.A.); 2 Disability Research Institute, Health Sector, King Abdulaziz City for Science and Technology (KACST), Riyadh 11442, Saudi Arabia; mkhuryef@kacst.gov.sa; 3 Bioengineering Institute, Health Sector, King Abdulaziz City for Science and Technology (KACST), Riyadh 11442, Saudi Arabia; mmajrashi@kacst.gov.sa; 4 Institute of Health Technologies and Preventive Medicine—Health Sector, King Abdulaziz City for Science and Technology (KACST), Riyadh 11442, Saudi Arabia

**Keywords:** nanoparticles, cancer immunotherapy, drug delivery

## Abstract

Cancer treatment has undergone a paradigm shift following the introduction of novel cancer treatment approaches that involve the host’s immune system in fighting established tumors. This new concept aids the immune system in identifying, attacking, and killing the tumor cells. However, although some encouraging results were observed clinically, this approach has its own limitations. For example, the benefits of certain anticancer drugs were only observed in some patients, off-target effects, immune evasion, and poor pharmacokinetics. Recently, several advancements have been made with the understanding and development of tumor-targeted drug delivery systems, which combine both effectiveness and patients’ safety during cancer treatment. In this review, we will focus on the latest progress in targeted drug delivery, particularly applying nanoparticles, liposomes, exosomes, and Wharton’s jelly-derived macrovesicles as immune cell enhancers, as well as overcoming therapeutic resistance. We also characterize major current problems, such as the biocompatibility and scalability of the delivered engineering systems, as well as the required regulations. Lastly, we will show some examples of effective approaches to resolve these issues for more efficient cancer therapy. The importance of this article lies in bridging two sides in a single framework perspective: the novel implementation of unique delivery systems and the latest advances in the field of cancer immunotherapy. Thus, this provides better insights for the future of cancer treatment.

## 1. Introduction

Cancer is still among one of the leading causes of death worldwide. According to the National Institutes of Health (NIH), in 2022, almost 20 million new cancer cases were reported, and around 10 million deaths were related to cancer [1]. The number of cancer-related deaths is expected to increase in the future to reach around 15 million by 2040 [1]. Cancer is a result of the abnormal proliferation of a particular type of cell in the body. We now have more than a hundred different types of cancer that have different behaviors and different responses to cancer drugs [2].

Although traditional cancer treatment approaches are showing some efficacy toward treating cancer patients, they are not always effective. Plus, drug-related adverse events have been reported with traditional cancer treatments [3]. Various adjuvants are currently used in cancer immunotherapy to stimulate an antitumor reaction, which includes cytokines, checkpoint inhibitors, and Toll-like receptor (TLR) agonists [4].

Cancer immunotherapy approaches, including monoclonal antibodies (mAbs), peptides, adoptive cell transfer, tumor-infecting viruses, cytokines, checkpoint inhibitors, Stimulator of Interferon Genes (STING), and Toll-Like Receptor (TLR) agonists, have been developed to induce robust antitumor immune responses [4] (This Ref from Dr.Nojoud Part). However, despite their potential effects, several considerable challenges seem to hinder their successful implementation.

The effectiveness of immunotherapy in treating cancer has been reported to be influenced by the tumor mutational burden (TMB). Higher levels of TMB indicate a greater number of mutations in cancer cells, which creates more neoantigens available for the immune system to recognize, leading to robust immune responses against the tumor and substantially improving the success of immunotherapy [1,5]. However, cancer cells are not inherently immunogenic; they can escape natural immune detection and killing. Additionally, the tumor microenvironment can actively suppress the immune response, impacting the T lymphocytes’ effector function and leading to dysfunctional antitumor immune responses [1]. This allows the tumor to progress unchecked and enables it to grow to be clinically detectable, and it becomes more difficult for the immune system to eradicate the tumor. Tumor heterogeneity also complicates cancer therapy, resulting in uneven treatment responses and treatment failure in some cases. Moreover, the adaptability of cancer signaling networks allows tumor cells to develop multiple resistance mechanisms to various cancer therapies over time, posing a significant challenge to achieving long-lasting treatment success. Thus, ongoing development in immunotherapy strategies is required to achieve the desired treatment outcomes [1].

The use of nanoparticles in cancer immunotherapy has emerged as a transformative platform due to their ability to improve drug delivery, target specific tumor cells, and modulate the immune system [6]. These nanoparticles have a size that ranges between 5 and 200 nm and can accumulate in cancer cells through the enhanced permeability and retention (EPR) effect [7]. Lipid-based nanoparticles have been investigated for many decades and have long been the most common drug delivery system in cancer therapy. These particles have structural characteristics that are similar to those of the plasma membrane of cells, which contribute to their favorable safety, simplicity, biocompatibility, degradability, drug loading capacity, and low toxicity [8]. These particles can target tumor cells by either a passive or active mechanism. Passive targeting relies on the tumor’s leaky vasculature with a pore size of 100–800 nm, allowing the penetration and accumulation of these particles in tumor cells [9,10]. On the other hand, active targeting focuses on functionalizing nanoparticles with ligands binding to specific receptors overexpressed on cancer cells to enhance specificity and selectivity. This can be achieved by decorating the surface of nanoparticles with tumor-specific ligands such as monoclonal antibodies (mAbs), peptides (RGD), folate, transferrin, and aptamers [5]. Additionally, nanoparticles can target immune cells, inducing a cascade of immune reactions and subsequently modulating the tumor microenvironment (TME) to enhance antitumor immunity.

(Alhassan Odah) The use of nanoparticles in cancer immunotherapy is growing enormously [1,3]. A number of scientists are currently focusing their research on understanding their biological systems and trying to understand and analyze their interaction with tumor cells to develop more successful anticancer drugs that can specifically target and kill tumor cells with no or minimal side effects [1,3].

## 2. Overcoming Drug Resistance Mechanisms

The tumor microenvironment (TME) consists of various cell types and other components, including immune cells, endothelial cells, and extracellular matrix, all of which play a major role in tumor development, progression, and establishing resistance mechanisms to multiple anticancer drugs [11]. Therefore, developing targeted drug delivery systems might be a promising alternative for cancer treatment.

Although traditional cancer therapies, particularly chemotherapy, have proven to be effective against multiple tumors throughout the past years, extended exposure to these toxic agents could result in the development of a defense mechanism known as multidrug resistance (MDR), ultimately contributing to cancer progression and poor recovery [12]. Multiple cellular and physiological factors have been shown to contribute to the development of MDR, such as the overexpression of ATP-binding cassette (ABC), transporters like P-glycoprotein (P-gp), multidrug resistance-associated proteins (MRPs), and breast cancer resistance protein (BCRP). All these factors play a critical role in hindering the efficacy of chemotherapy [13]. Plus, mutations or loss of function of the p53 tumor suppressor gene can disrupt apoptosis and increase the expression of anti-apoptotic proteins like the B-cell lymphoma 2 (Bcl-2) protein [14,15]. In addition to the cellular factors, there are also physiological factors that can also directly contribute to the tumor growth, including the presence of tumor-associated fibroblasts (TAFs), which secrete protective cytokines and extracellular matrix components; high interstitial fluid pressure (IFP), which impairs the antitumor drug penetration into the tumor; and finally, the acidic and hypoxic tumor microenvironment, which alters drug activity and uptake [13,14].

Advancements in nanotechnology may serve as a promising approach in improving the efficacy of chemotherapy. Results from multiple studies showed great potential in overcoming various MDR mechanisms. Below, we will discuss some of the nanotechnology approaches that have been developed and their applications in counteracting MDR pathways to eradicate tumors and enhance therapeutic outcomes.

### 2.1. Targeted Drug Delivery to Tumor Hypoxia

Most solid tumor cells exist in a hypoxic microenvironment, which is considered a key contributor to chemotherapy resistance [16]. This oxygen-deficient microenvironment results from the rapid proliferation of tumor cells and the formation of irregular blood vessels. Since acidosis occurs in the absence of oxygen, modulating the hypoxic microenvironment and the acidity of the TME could inhibit chemotherapy drug resistance [17]. One approach that can be applied to mitigate tumor hypoxia is the specific delivery of oxygen ions into the tumor site by using nanocarriers. Parsad et al. developed a polyelectrolyte–albumin complex coupled with MnO_2_ nanoparticles (A-MnO_2_ NPs) to attenuate hypoxia and acidosis in the TME; the study showed that the NP was able to generate O_2_ by reacting with H_2_O_2_ under hypoxic conditions, while the pH within the tumor was increased from 6.7 to 7.2 [18]. During the hypoxia process, the overexpression of hypoxia-inducible factor 1α (HIF-1α) (which is known to promote tumor cell growth) has been observed in different human cancers. Therefore, targeting HIF-1α could be a promising strategy to overcome hypoxia and to improve tumor sensitivity to chemotherapy [19,20]. Plus, it has been shown that the delivery of small interfering RNA (siRNA) robustly blocks tumor cell progression by silencing the HIF-1α/CD73 axis. This can be achieved by using siRNA-loaded TAT-chitosan-SPION nanoparticles, which significantly reduced the expression levels of HIF-1α and CD73 in tumor cells (<25% compared to untreated cells), leading to decreased migration, proliferation, and tumor growth [21].

### 2.2. Targeted Delivery to Efflux Transporters

Efflux transporters belong to the mammalian adenosine triphosphate (ATP)-binding cassette (ABC) family of transporters. They have been reported to play a key role in chemotherapy resistance [22]. After the drug is internalized through the cell’s plasma membrane, cellular transporters rapidly recognize and expel the drug out of the cell [12]. Several transporters that contribute to chemotherapy resistance have been identified in humans, particularly P-glycoprotein (P-gp), which is widely expressed in several MDR tumors [23]. Various strategies were employed to enhance drug availability by counteracting the effects of efflux transporter proteins, including the use of efflux pump inhibitors, drug modification to evade recognition, and encapsulation within nanocarriers to bypass transporter-mediated efflux. Therefore, multiple nanoparticles (NPs) were developed to specifically target cancer cells to bypass the cell membrane and to deliver the cargo to the cytoplasm away from the efflux pump [24,25,26]. In addition, combination therapy has been proposed as a strategy to overcome MDR by co-delivering efflux pump inhibitors alongside chemotherapy [14]. For example, the delivery of miR495 using silica NPs was significantly more effective in overcoming MDR by downregulating P-glycoprotein (P-gp) expression, leading to an enhanced intracellular drug accumulation and significantly decreased MDR of lung cancer cells to doxorubicin (A549/DOX) to 38.5% compared to 80% survival when cells were treated with free doxorubicin [27]. Moreover, the co-delivery of Cyclooxygenase-2 (COX-2), a P-gp inhibitor, with doxorubicin (DOX) significantly increased the cytotoxicity effect of DOX chemotherapy on MCF-7/ADR cell lines [28]. Moreover, in MCF-7/ADR tumor-bearing nude mice, nanoparticles demonstrated superior tumor-targeting capability and distribution, significantly increased tumor size reduction, and decreased COX-2 and P-glycoprotein expression in tumor tissues by less than 0.5-fold.

## 3. Harnessing Nanoparticles for Immune Cell Activation

Various immune cells, including macrophages, neutrophils, and lymphocytes, are recruited in the TME in response to the chemokines and cytokines that are secreted by the TME [29]. When these cells become activated, they secrete various cytokines and chemokines [30]. However, persistent activation of the immune cells and the inability to resolve the inflammatory response contribute to chronic inflammation. Thus, this facilitates tumor growth, invasion, and metastasis [30,31].

Macrophages are one of the most active immune cells during tumorigenesis. These cells exhibit remarkable phenotypic and functional diversity in response to their surrounding environment, adopting either a pro-inflammatory (M1) phenotype or an anti-inflammatory (M2) phenotype [32]. M1 macrophages play a crucial role in enhancing tumor cell cytotoxicity, either directly by releasing cytotoxic molecules such as reactive oxygen species (ROS) and nitric oxide (NO), or indirectly by activating natural killer (NK) and cytotoxic T cells through cytokine signaling (e.g., interferon-gamma (IFN-γ) and tumor necrosis factor alpha (TNF-α)) [33,34]. Moreover, M1 contributes to the activation of adaptive antitumor responses, particularly T cells, via antigen presentation and pro-inflammatory cytokine secretion, such as IL-12 and TNF-α, to stimulate T-cell proliferation [31,34,35]. TME is dominated by M2 macrophages, on the other hand, which suppress the efficacy of M1 cytotoxicity and promote tumor progression by secreting high levels of cytokines, such as interleukin-10 (IL-10) and transforming growth factor beta (TGF-β) [35]. Several immunotherapeutic molecules were introduced to tackle tumor cells, including mAbs, small molecules, peptides, and proteins. However, the delivery of such therapeutic agents remains challenging due to the following reasons: poor targetability, permeability through cancer cells, and uptake by phagocytic cells [36]. NPs hold a promising solution to overcome the aforementioned challenges [11]. For example, nanoparticles have been employed to selectively target and modulate TAMs, promoting their polarization from the M2 (protumor) to M1 (antitumor) phenotype. Several studies demonstrated that nanoparticles functionalized with mannose or hyaluronic acid (HA) can selectively bind to TAMs via the mannose receptor, triggering immune activation and enhancing the antitumor immune response. Song et al. developed mannose-modified (HA)-coated MnO_2_ nanoparticles (Man-HA-MnO_2_ NPs) to target TAMs selectively. The study demonstrated that Man-HA-MnO_2_ NPs significantly increased the expression of inducible nitric oxide synthase (iNOS), a marker of M1 macrophages, while reducing CD206, which is a marker for M2 macrophages [37]. In another study, gold nanoparticles (AuNPs) and silver nanoparticles (AgNPs) showed their ability to directly induce the M1-like polarization of TAMs by increasing reactive oxygen species (ROS) and reactive nitrogen species (RNS) production [38]. Moreover, Wang et al. developed IL-12-loaded poly (beta-amino ester) nanoparticles (IL-12@P1 NPs) designed specifically to reprogram TAMs in acidic TME conditions. In vitro, studies showed that IL-12@P1 NPs effectively converted M2-polarized RAW264.7 macrophages into the M1 phenotype. In vivo studies, on the other hand, demonstrated that IL-12@P1 NPs preferentially accumulated in the tumor, resulted in significantly enhanced antitumor immunity, and inhibited approximately 80% of tumor growth compared to the control groups that were treated with free IL-12, nanoparticles alone, or PBS [39].

### 3.1. Innovations in Non-Lipid-Based Nanoparticle Drug Delivery

The field of targeted drug delivery has significantly evolved with the integration of nanotechnology and biomaterials, enhancing the precision and efficacy of cancer immunotherapy. These advanced delivery platforms ensure selective drug accumulation at tumor sites while minimizing systemic toxicity. This section discusses key innovations, particularly non-lipid-based nanoparticles, and their role in improving therapeutic outcomes. Advancements in nanotechnology have enabled the development of stimuli-responsive delivery systems that release their payload in response to specific tumor microenvironmental cues such as pH, redox potential, or enzymatic activity [40]. Additionally, hybrid nanoparticle systems are being explored to combine the advantages of multiple platforms, offering enhanced drug loading capacity, controlled release kinetics, and improved biocompatibility [40]. Non-lipid-based polymeric, inorganic, carbon-based, and magnetic nanoparticles have enabled precise drug delivery while mitigating systemic toxicity [41]. The optimization of these technologies for clinical translation by addressing biocompatibility, large-scale manufacturing, and regulatory hurdles would mark a crucial step toward more effective and personalized cancer immunotherapy strategies.

### 3.2. Engineering Considerations for Optimized Drug Targeting

Advancements in nanotechnology have significantly transformed the landscape of cancer immunotherapy, mainly through the engineering of stimuli-responsive capabilities [42]. These nanosystems can be finely tuned to respond to specific features of the tumor microenvironment (TME), including the solid tumor’s acidic pH, elevated glutathione (GSH) levels, and overexpressed enzymes such as matrix metalloproteinases (MMPs) [43]. For example, pH-sensitive polymeric nanoparticles release their therapeutic payload upon encountering the acidic conditions typical of the tumor interstitium (pH ~6.5). At the same time, redox-sensitive carriers exploit the intracellular GSH concentration gradient to trigger payload release within tumor cells [43].

Functionalization with targeting ligands such as monoclonal antibodies (e.g., anti-HER2), peptides (e.g., RGD), folic acid, and transferrin enhances selective accumulation in tumors that overexpress the corresponding receptors [44]. A noticeable example is folate-functionalized poly(lactic-co-glycolic acid) (PLGA) nanoparticles, which have demonstrated up to a 5-fold increase in cellular uptake in folate receptor-overexpressing ovarian cancer cells relative to non-targeted controls [45]. Furthermore, polymeric systems enable combination therapies that co-deliver chemotherapeutic agents and immune modulators. For instance, in breast cancer models, PLGA nanoparticles co-encapsulating paclitaxel and anti-CTLA-4 antibodies demonstrated a 50% greater tumor regression rate than either agent alone, indicating a powerful synergy between cytotoxic and immunomodulatory mechanisms (Table 1) [42]. Despite these advances, challenges remain in optimizing nanoparticle stability, minimizing premature drug leakage, ensuring manufacturing reproducibility, and achieving selective biodistribution to avoid off-target effects [46,47,48]. Nonetheless, with continued progress in polymer chemistry, nanofabrication, and immunoengineering, polymeric nanoparticles offer a highly modular and effective strategy to enhance cancer immunotherapy’s specificity, potency, and personalization.

#### 3.2.1. Polymeric Nanoparticles: Versatile and Biocompatible Carriers

Polymeric nanoparticles have emerged as a powerful platform for targeted drug delivery, capable of encapsulating a diverse range of therapeutic agents, including small molecules, proteins, and nucleic acids [49]. Their highly tunable physicochemical properties enable precise control over particle size, surface charge, and drug release kinetics, making them well suited for cancer immunotherapy applications [49].

Biodegradable polymers such as PLGA, polylactic acid (PLA), and polyethylene glycol (PEG) are widely used in nanoparticle formulations due to their favorable safety profiles [50,51]. Among these, PLGA has received FDA approval due to its excellent biocompatibility and controlled degradation into lactic and glycolic acid, which are naturally metabolized by the body [50]. Depending on the formulation parameters, PLGA nanoparticles can sustain drug release over durations ranging from hours to several weeks, providing a controlled and prolonged therapeutic effect [50,52].

A wide array of polymers, both synthetic and natural, have been approved or recognized by the FDA for use in cancer drug delivery, with approximately over 20 polymers currently utilized in various clinical and experimental formulations [53]. These include biodegradable carriers like PLGA and PLA; surface modifiers such as PEG and poloxamers; and biologically derived materials like chitosan, hyaluronic acid, and gelatin [54]. Their roles span from forming nanoparticle matrices and hydrogels to enabling targeted delivery, sustained release, and immune modulation. Several polymers, such as PLGA in Lupron Depot^®^ (Abbvie Endocrine, North Chicago, IL, USA) and PEG in Doxil^®^ (Johnson and Johnson, New Brunswick, NJ, USA), have historically achieved clinical success, while others are advancing through preclinical and clinical trials for targeted chemotherapy and chemo-immunotherapy [55]. Ongoing innovation in polymer chemistry continues to improve the efficacy, safety, and specificity of cancer nanomedicines. In the following section, some of the prominent polymers are discussed in more detail.

##### Poly(ε-caprolactone) (PCL)

In addition to these commonly used polymers, several other biodegradable and biocompatible polymers have been explored for drug delivery applications. PCL is a semicrystalline, biodegradable polyester known for its biocompatibility, hydrophobic nature, and slow degradation rate, making it an excellent candidate for long-term drug delivery. It primarily degrades through the hydrolytic cleavage of ester bonds, where water molecules break the ester linkages in the polymer backbone, leading to the gradual erosion of PCL into 6-hydroxycaproic acid, which is further metabolized via the Krebs cycle [1]. This property enables sustained drug release, as demonstrated by PCL-based formulations encapsulating paclitaxel or antibodies, which maintained drug release for several days to weeks, depending on the formulation (Table 1) [56,57,58]. In addition, PCL can be processed into various biomedical forms, such as nanoparticles, microspheres, hydrogels, and scaffolds, allowing for controlled and prolonged drug delivery [56,58,59].

Poly(ε-caprolactone) (PCL)-based polymeric nanoparticles have been extensively studied on various cancer cell lines, including the murine triple-negative breast cancer cell line (4T1), human ovarian cancer cells (SKOV3), and colorectal adenocarcinoma (Caco-2) cells, demonstrating enhanced cellular uptake, sustained drug release, and improved cytotoxicity, particularly when combined with targeting ligands or immunomodulatory agents [1,2,3].

PCL compatibility with polymers such as PLGA and PEG enables the customization of physicochemical properties to meet specific application requirements. Blending PCL with PLGA allows for the modulation of mechanical properties and degradation rates. PLGA’s higher stiffness and faster degradation balance PCL’s flexibility and slower biodegradation, resulting in materials with tailored characteristics suitable for drug delivery and tissue engineering [60]. Similarly, incorporating PEG into PCL matrices enhances hydrophilicity and facilitates tuning mechanical properties and degradation rates. PCL-PEG-PCL triblock copolymers, synthesized via ring-opening polymerization, have demonstrated adjustable elastic moduli ranging from 338 to 705 megapascal (MPa) and degradation rates from 60% mass loss after 8 h to 70% after 23 days in accelerated tests while maintaining excellent cytocompatibility [61]. These modifications enable the development of hybrid biomaterials with improved bioadhesion, controlled drug release kinetics, and enhanced cellular interactions, thereby broadening the scope of PCL-based systems in biomedical fields.

Despite its advantages, PCL has several limitations that can restrict its applications. Its slow degradation rate, while beneficial for prolonged drug release, can be a drawback for drugs requiring rapid clearance from the body [62]. Additionally, PCL’s hydrophobic nature often results in poor dispersibility in aqueous environments, limiting its effectiveness for hydrophilic drug delivery, unless it is modified with hydrophilic polymers such as PEG [62]. Another challenge is its low bioactivity, as unmodified PCL lacks functional groups that promote strong cellular interactions, which can hinder its performance in tissue engineering [63].

##### Chitosan

Chitosan, a cationic polysaccharide derived from chitin, has emerged as a versatile nanomaterial in cancer immunotherapy due to its biocompatibility, biodegradability, and ability to enhance immune responses [64]. Its unique properties, including mucoadhesion and the capacity to form nanoparticles, enable effective delivery of therapeutic agents. Chitosan nanoparticles have been extensively explored for gene delivery applications, achieving transfection efficiencies up to 80% in certain cancer cell lines, thereby facilitating the introduction of tumor-suppressing genes and immunostimulatory molecules to enhance antigen presentation and stimulate robust antitumor immune responses. Additionally, chitosan-based nanoparticles have been utilized for the targeted delivery of chemotherapeutic agents, improving drug solubility, bioavailability, and therapeutic efficacy while minimizing systemic toxicity (Table 1) [65]. For instance, the encapsulation of docetaxel into chitosan nanoparticles has demonstrated enhanced anticancer efficacy against breast cancer cells, with in vivo studies showing a tumor volume reduction to 260 mm^3^ compared to 415 mm^3^ for uncoated SLNs and 600 mm^3^ for free docetaxel, reflecting a 57% and 37% greater tumor inhibition, respectively (*p* < 0.05) [66,67]. Recent advancements in cancer immunotherapy have led to the development of chitosan-coated hollow copper sulfide nanoparticles (HCuSNPs) integrated with immunoadjuvants, demonstrating enhanced therapeutic efficacy against both primary and distant tumors. These nanoparticles are designed to deliver oligodeoxynucleotides containing cytosine-guanine motifs (CpG ODNs), which serve as Toll-like receptor 9 (TLR9) agonists, stimulating immune responses [30]. Upon near-infrared (NIR) laser irradiation, the HCuSNPs exhibit photothermal properties that induce localized tumor cell death, releasing tumor-associated antigens that facilitate a sufficient antitumor immune response. Concurrently, heat triggers the disintegration of HCuSNPs, leading to the formation of chitosan-CpG nanocomplexes that enhance tumor retention and uptake by dendritic cells (DCs). This combined photothermal and immunotherapeutic approach has shown superior outcomes to monotherapy [68]. The study demonstrates that chitosan-coated hollow copper sulfide nanoparticles combined with CpG and near-infrared laser irradiation elicit up to a 41-fold increase in immune activation, a 19-fold enhancement in systemic CD8^+^ T-cell response, complete suppression of distant tumor growth for 10 days, and over 47% nanoparticle clearance within 14 days, confirming potent, systemic, and biodegradable photothermal immunotherapy efficacy [68]. These findings highlight the potential of HCuSNPs as a multifunctional platform for effective cancer therapy [69]. These multifaceted applications underline chitosan’s potential as a nanomaterial in cancer immunotherapy, offering avenues for gene delivery, targeted chemotherapy, and combination therapies that enhance immune responses against tumors.

#### 3.2.2. Poly(alkyl cyanoacrylate) (PACA)

Poly(alkyl cyanoacrylate) (PACA) nanoparticles, composed of polymerized alkyl cyanoacrylate monomers with the general chemical structure [-CH_2_-C(CN)(COOR)-]_n_, where R denotes an alkyl group such as butyl or ethyl, have emerged as a promising nanocarrier system in cancer immunotherapy due to their rapid enzymatic degradation, high drug-loading capacity, and exceptional ability to cross biological barriers, particularly the blood–brain barrier (BBB) [70,71]. The degradability of PACA is attributed to the hydrolytic cleavage of the ester bond in the side chain, generating non-toxic alcohols and cyanoacetic acid, which are readily cleared from the body [34]. Notably, poly(butyl cyanoacrylate) (PBCA) nanoparticles coated with polysorbate 80 have shown a remarkable enhancement in drug delivery to the brain. For instance, doxorubicin-loaded PBCA nanoparticles increased brain accumulation of the drug by up to 60-fold compared to free doxorubicin, enabling the effective treatment of intracranial gliomas in preclinical models [72,73]. This enhanced delivery is likely mediated by the adsorption of apolipoproteins from the bloodstream onto the nanoparticle surface, facilitating receptor-mediated transcytosis across the BBB [73]. From an immunotherapeutic perspective, PACA nanoparticles can be engineered to deliver immune checkpoint inhibitors (e.g., anti-PD-1/PD-L1 antibodies), tumor-associated antigens, or immune adjuvants, thereby promoting antigen presentation and T-cell activation (Table 1). For example, PACA nanoparticles co-loaded with tumor antigen peptides have demonstrated the potent activation of DCs and the induction of cytotoxic T lymphocyte (CTL) responses in melanoma-bearing mice [74]. Moreover, PACA nanoparticles’ rapid degradation can reduce the amount of time that immune cells are exposed to synthetic polymers, potentially lowering immunotoxicity [74]. Collectively, PACA offers flexible and adjustable nanoplatforms that can connect immunotherapeutic and chemotherapeutic strategies, especially for resistant tumors such as glioblastoma and metastatic brain cancers.

##### Poly(glutamic acid)

Poly(γ-glutamic acid) (PGA) is a naturally occurring, biodegradable, and water-soluble polypeptide made up of repeating glutamic acid units connected by γ-amide bonds ([-CO-CH(NH_2_)-(CH_2_)_2_-COO-]_n_). Because of its excellent biocompatibility, tunable molecular weight, and numerous carboxylic acid side chains that aid in drug conjugation or encapsulation, PGA has shown great promise as a nanocarrier in cancer immunotherapy [75]. PGA-based nanoparticles have been particularly effective in enhancing the therapeutic index of cytotoxic agents by improving tumor targeting and minimizing off-target toxicity. One noticeable example is poly(γ-glutamic acid)-cisplatin (PGA-CDDP) conjugates, which form self-assembling nanoparticles that exploit the enhanced permeability and retention (EPR) effect to selectively accumulate in tumor tissues [76]. In preclinical settings, PGA-CDDP nanoparticles maintained or enhanced antitumor activity while achieving a 2.5-fold increase in tumor accumulation when compared to free cisplatin and a significant reduction in nephrotoxicity and systemic adverse effects [76]. In terms of immunotherapy, PGA nanoparticles have also been used to deliver immune modulators—like cytokines and TLR agonists—directly to the tumor microenvironment. For instance, PGA coupled with the TLR7 agonist imiquimod has demonstrated effective DC activation and systemic antitumor immune response in melanoma mouse models (Table 1) [77]. By encouraging DC maturation and upregulating co-stimulatory molecules, including CD80 and CD86, PGA has been demonstrated to directly stimulate DCs, improving antigen presentation and subsequent T-cell activation [78]. Similarly, PGA’s anionic nature makes it easier to combine with cationic antigens or adjuvants, which makes it a potential platform for delivering cancer vaccines [75]. PGA-based nanogels and micelles have also been investigated recently for the co-delivery of immunotherapeutic and chemotherapeutic drugs, offering synergistic effects by stimulating T-cell infiltration and immunogenic cell death [78]. With its immunomodulatory, biodegradable, and modifiable properties, PGA is a promising multifunctional nanocarrier in the developing field of cancer immunotherapy.

**Table 1:** Polymeric nanoparticles and their effects on cancer cell lines.

Polymeric Nanoparticle	Encapsulated Agent	Cancer Cell Line (s)	Observed Effect	References
PLGA	mSTEAP peptide	Adenocarcinoma mouse prostate (TRAMP)-C2 cells, melanoma cell lines (624 and 1351), and human tumor-infiltrating lymphocytes	Synergistic tumor regression (~50% increase over monotherapy)	[4,42]
PLGA (folate-functionalized)	Doxorubicin	OVCAR-3 (ovarian)	5-fold increase in uptake due to folate receptor targeting	[45]
PCL (Poly(ε-caprolactone))	Paclitaxel, antibodies	MCF-7 (breast), A549 (lung)	Sustained drug release, enhanced cytotoxicity, tumor suppression	[57,58]
Chitosan	Docetaxel	MCF-7 (breast)	57% greater tumor inhibition; improved drug delivery	[66,67]
PACA (Polyalkyl cyanoacrylate)	Doxorubicin, tumor peptides	Patient-derived basal-like xenograft (MAS98.12)	Increased ratio of M1/M2 (antitumorigenic/protumorigenic) macrophages in the tumors	[6,72,73]
PGA (Poly(γ-glutamic acid))	Cisplatin, Imiquimod	B1F10 (melanoma) and bone marrow (BM)-derived DCs (BMDCs)	2.5-fold tumor accumulation, reduced nephrotoxicity, enhanced DC activation	[76,77,78]

#### 3.2.3. Inorganic Nanoparticles: Precision and Multifunctionality

By combining nanotechnology with biomaterials, the science of targeted drug delivery has made substantial progress, greatly increasing the accuracy and effectiveness of cancer immunotherapy. Due to their high stability, variable size, and ease of functionalization, non-lipid-based nanoparticles, especially inorganic forms like gold nanoparticles (AuNPs), mesoporous silica nanoparticles (MSNs), and quantum dots (QDs), have shown remarkable promise among evolving techniques [79]. These multifunctional platforms enable selective drug accumulation at tumor sites while minimizing systemic toxicity, and they also support applications in imaging and immunomodulation, positioning them as key components in next-generation cancer treatment approaches.

#### 3.2.4. Gold Nanoparticles (AuNPs)

Due to their distinct physicochemical and optical characteristics, gold nanoparticles (AuNPs) have become multipurpose platforms in cancer immunotherapy, primarily through their use in immune regulation and photothermal treatment (PTT) [80,81]. Structurally, AuNPs are composed of elemental gold (Au^0^), typically synthesized in nanoscale diameters ranging from 1 to 100 nm and functionalized with surface ligands for stability and targeting [82]. They can effectively absorb near-infrared (NIR) light (650–900 nm) and transform it into localized heat for tumor ablation, thanks to their strong surface plasmon resonance [80]. In addition to directly killing cancer cells, this heat-induced disruption, or PTT, also stimulates immunogenic cell death (ICD), which increases the release of tumor-associated antigens (TAAs) and danger-associated molecular patterns (DAMPs), which in turn helps DC maturation and cytotoxic T lymphocyte activation [83,84,85]. For instance, in melanoma-bearing mice, gold nanoparticles (AuNPs) that co-delivered CpG oligonucleotides and ovalbumin (OVA), a model tumor-associated antigen, significantly improved dendritic cell (DC) maturation and stimulated strong antigen-specific CD8^+^ T-cell responses. This nanovaccine method led to 60–70% tumor growth suppression and a 3- to 4-fold increase in CD8 T-cell infiltration in vivo [86]. These results highlight the multifunctional potential of AuNPs as immunotherapeutic platforms for effective cancer vaccination strategies [87].

In addition to PTT, AuNPs have been used in testing as precise delivery systems for immune checkpoint inhibitors, including anti-PD-1 and anti-CTLA-4 antibodies, which enhance tumor selectivity and lower systemic toxicity [88]. Gold nanoparticles functionalized with PD-L1 inhibitors, such as anti-PD-L1 antibodies or PD-L1-binding peptides, have been shown in preclinical studies to greatly increase CD8^+^ T-cell infiltration and improve tumor control when compared to free PD-L1 inhibitors [89]. For example, AuNP-based delivery enhanced therapeutic efficiency in colorectal and breast cancer models by enabling targeted tumor accumulation and stimulating immune activation in the tumor microenvironment [90,91]. These results highlight the ability of gold nanocarriers tailored to PD-L1 to enhance immune checkpoint blockage while reducing systemic toxicity. Furthermore, AuNPs enable strong adaptive immune responses by providing flexibility for the co-delivery of cytokines, adjuvants, and antigens. Their inert gold core ensures biocompatibility and low immunogenicity, while their large surface area-to-volume ratio permits the multivalent presentation of ligands [92,93,94]. Gold nanoparticles could provide a potent platform for cancer immunotherapy, integrating targeted tumor ablation with immune system activation to elicit durable antitumor responses.

##### Silver Nanoparticles (AgNPs)

Silver nanoparticles (AgNPs), widely used in products like wound dressings and diabetic socks due to their antibacterial, antifungal, and anti-inflammatory properties, have demonstrated clinical utility. However, their potential cytotoxicity at high concentrations poses a risk to mammalian cells, necessitating careful application [95]. Recent preclinical studies have highlighted the potential of AgNPs in cancer immunotherapy, particularly those ~5 nm in size with citrate or PVP surface coatings [96]. Citrate-coated AgNPs at 5 µg/mL induced >60% cytotoxicity in murine renal carcinoma (Renca), with a ~4-fold increase in mitochondrial ROS and significant calreticulin translocation—key hallmarks of immunogenic cell death (ICD) [97,98,99]. The intratumoral peritumoral injections of 20 µg Ag-citrate-5 nm combined with 150 µg anti-PD-1 (three doses, 4-day intervals) led to a 70% reduction in tumor bioluminescence and a 2- to 3-fold increase in CD8^+^ T-cell infiltration compared to either monotherapy [97]. At higher dosing (50 µg AgNP + 200 µg anti-PD-1), tumor suppression improved further, with enhanced calreticulin exposure and systemic CD69^+^CD8^+^ T-cell activation in splenocytes [97]. Additionally, cytokine profiling revealed dose-dependent upregulation of IL-6, IFN-α/β, IFN-γ, IL-12, and TNF-α following 5 µg/mL AgNP exposure [97]. Complementary biosyntheses, such as aloe vera-derived AgNPs targeting MCF-7 cells, have shown robust in vitro cytotoxicity, further supporting AgNP versatility [11]. These results collectively demonstrate that ultrasmall, surface-tuned AgNPs can trigger potent ICD, reshape the tumor microenvironment, and synergize with the checkpoint blockade, justifying further investigation into their dosing regimens, biodistribution, and translational potential.

#### 3.2.5. Mesoporous Silica Nanoparticles (MSNs)

Mesoporous silica nanoparticles (MSNs), which are mostly made of silicon dioxide (SiO_2_) and have a highly organized, porous structure, have become effective nanocarriers in cancer immunotherapy because of their high surface area (>1000 m^2^/g) and great biocompatibility [100]. The repeating -[SiO_4_]-network that makes up MSNs’ chemical structure forms a stiff, porous lattice that is easily functionalized for targeted drug administration. [100,101]. Due to these characteristics, MSNs are especially useful for the co-administration of immunomodulators and chemotherapeutic drugs, allowing for the spatiotemporal regulation of simultaneous drug release to promote therapeutic synergy [100]. For example, a preclinical study showed that in a mouse breast cancer model, lipid-coated biodegradable hollow MSNs co-loaded with interleukin-2 (IL-2), doxorubicin (DOX), and all-trans retinoic acid (ATRA) markedly improved antitumor immunity. Tumor growth and metastasis were significantly inhibited by this combinatorial approach, which increased cytokine secretion (e.g., IFN-γ and IL-12), suppressed immunosuppressive elements within the tumor microenvironment, and promoted infiltration and activation of tumor-infiltrating lymphocytes (TILs), including T cells and natural killer cells [102]. In addition to drug delivery, MSNs have been shown to actively modulate the TME. For example, MSNs carrying ROS-generating agents such as manganese dioxide or photosensitizers can induce the oxidative stress within tumors, triggering immunogenic damage and enhancing DC recruitment and antigen presentation [102].

Moreover, the surface functionalization of MSNs with targeting ligands like HA can improve selective binding to CD44 receptor-overexpressing tumor cells, thus enhancing cellular uptake and minimizing off-target toxicity [103]. This targeted approach not only improves treatment precision but also helps to preserve surrounding healthy tissue. Due to their flexible design, MSNs can also be adapted for combination therapies involving checkpoint inhibitors, cancer vaccines, or adjuvants, further strengthening their value in immuno-oncology [104,105,106]. Overall, MSNs could be a powerful and flexible platform for enhancing the efficacy and specificity of cancer immunotherapy through controlled, multi-agent delivery and active modulation of the immunosuppressive TME.

#### 3.2.6. Quantum Dots (QDs)

Quantum dots (QDs) are nanoscale semiconductor crystals—typically composed of elements such as cadmium selenide (CdSe), cadmium telluride (CdTe), or indium phosphide (InP)—encapsulated within a core-shell structure (e.g., CdSe/ZnS) and offering distinctive optical properties due to quantum confinement effects [107]. Their chemical makeup and adjustable size-dependent emission spectra allow for accurate fluorescence imaging over a wide wavelength range, which makes them ideal for high-resolution, real-time visualization of immune cell infiltration, drug biodistribution, and tumor growth in vivo [108]. QDs are now used in cancer immunotherapy in addition to their conventional use in diagnostics. They have been engineered as theranostic platforms for the co-delivery of therapeutic payloads, including small interfering RNA (siRNA), immune adjuvants, and tumor antigens [109,110]. For instance, QDs conjugated with siRNA have demonstrated effective gene silencing in lung, breast, and brain cancer models, achieving up to 70% mRNA inhibition in vitro [109]. Quantum dot-mediated siRNA delivery in cancer cells resulted in 50–85% gene knockdown, 30–80% tumor growth inhibition, and up to 4-fold increased cytotoxicity in combination therapies, primarily through targeted gene silencing (e.g., Survivin, TERT, HIF-1α, Bcl-2) and enhanced apoptosis [109]. These results highlight the feasibility of QD-mediated siRNA delivery to enhance antitumor immune responses and reduce tumor cell viability [111,112,113]. Additionally, QDs, especially cadmium-based core-shell structures like CdSe/ZnS, allow for the multiplexed and highly sensitive detection of tumor biomarkers such as hormone receptors, microRNAs, and HER2, which aids in the early detection of cancer and molecular stratification [114]. The QDs’ high quantum yield, narrow emission spectra, and resistance to photobleaching enable the high-resolution, real-time imaging of tumor growth and metastasis, even in aggressive subtypes like triple-negative breast cancer (TNBC), where traditional diagnostic techniques frequently fail [114]. Despite these developments, there is still limited clinical translation of QDs because of concerns about the long-term toxicity of their heavy metal components, including bioaccumulation, ROS production, and heavy metal ion release [115]. In response, new systems have focused on heavy metal-free QDs (such as carbon, silicon, or graphene quantum dots) that maintain photostability and fluorescence while enhancing safety profiles, as well as surface modification techniques employing biocompatible polymers (like PEGylation) or silica shells to reduce cytotoxicity [116,117,118]. With the growing recognition of imaging-guided immunotherapy, QDs present a solution for synchronized diagnosis, real-time monitoring, and immunotherapeutic drug delivery, with great potential for precision oncology in the future.

#### 3.2.7. Carbon-Based Nanoparticles: Emerging Platforms for Drug Delivery

The potential of carbon-based nanomaterials, including fullerenes, graphene oxide (GO), and carbon nanotubes (CNTs), in cancer immunotherapy is being investigated [119]. The distinct structural, electrical, and optical characteristics of these nanomaterials allow for targeted administration, effective drug loading, and TME manipulation to boost immune response [119].

#### 3.2.8. Graphene Oxide (GO) Nanoparticles

Because of their remarkable surface area, tunable chemistry, and synergistic photothermal and immunomodulatory properties, graphene oxide (GO) nanoparticles, which are made up of single-atom-thick sheets of carbon arranged in a two-dimensional hexagonal lattice with oxygen-containing functional groups (such as hydroxyl, carboxyl, and epoxide), have gained attention as a versatile platform in cancer immunotherapy [120]. Combinatorial immunotherapy techniques could benefit greatly from the GO’s high density of oxygenated groups, which enable the covalent or non-covalent conjugation of a variety of biomolecules, such as tumor antigens, adjuvants, and immune checkpoint inhibitors [120,121]. In order to improve local drug concentration and reduce systemic toxicity, GO nanoparticles have shown strong efficacy in boosting immune responses by delivering checkpoint inhibitors, such as anti-PD-L1 and anti-CTLA-4 antibodies, directly to the TME [121,122].

Furthermore, it has been demonstrated that GO coupled with Toll-like receptor (TLR) agonists, such as R848, can activate macrophages and dendritic cells, thereby improving antigen presentation and priming CTLs for a long-lasting antitumor response [1]. GO also has strong near-infrared (NIR) absorbance and can be used in PTT, where localized heating upon NIR irradiation induces immunogenic cell death, promoting the release of tumor-associated antigens and DAMPs [121,123]. In models of melanoma, breast, and colon cancer, the combination of GO-mediated PTT with checkpoint inhibitor therapy has shown synergistic effects, including increased immune cell infiltration and tumor shrinkage [124,125]. In order to enhance pharmacokinetics and lessen off-target toxicity, PEGylation and other surface engineering and modification methods are also being used to optimize the biocompatibility and modifiability of GO [126]. GO nanoparticles could be an excellent platform for enhancing the efficacy, precision, and durability of cancer immunotherapy through combinatorial delivery and immunogenic stimulation.

#### 3.2.9. Carbon Nanotubes (CNTs)

The unique physicochemical properties of carbon nanotubes (CNTs)—cylindrical nanostructures made of carbon atoms arranged in a hexagonal lattice (chemical structure: rolled-up sheets of graphene)—like high aspect ratio, large surface area, and inherent ability to translocate across cellular membranes, have attracted attention for cancer immunotherapy [127]. Because of these features, CNTs could be ideal nanocarriers for delivering immunotherapeutic drugs such as adjuvants, immunological checkpoint modulators, and tumor-associated antigens [128]. Functionalized CNTs, particularly single-walled (SWCNTs) and multiwalled (MWCNTs), can be chemically modified to improve solubility, biocompatibility, and targeting specificity [129]. For instance, MWCNTs functionalized with folic acid and coupled with chemotherapeutic drugs like methotrexate and doxorubicin greatly increased the selective uptake by cancer cells through endocytosis mediated by the folate receptor. This focused strategy allowed for enhanced cellular internalization and decreased systemic toxicity and produced a noticeable tumor shrinkage in preclinical animals, with strong CTL activation and increased antitumor efficacy [130]. Additionally, CNTs have been investigated for photothermal immunotherapy applications, where NIR irradiation of CNT-loaded tumors could induce ICD, further enhancing the antitumor immune responses [131,132]. Despite these promising results, concerns remain regarding the long-term persistence and potential toxicity of CNTs, requiring additional research into their biodegradation and clearance mechanisms [133,134].

#### 3.2.10. Fullerenes: Antioxidant and Immunomodulatory Properties

Fullerenes are a special class of carbon allotropes made up of carbon atoms arranged in hollow, spherical, closed-cage structures (often referred to as C_60_ or C_60_). They have unique physicochemical characteristics, such as high structural stability, strong electron affinity, and the capacity to scavenge radicals, which make them attractive options for cancer immunotherapy. Fullerenes’ spherical shape permits π-electrons to delocalize widely, which makes them powerful antioxidants that effectively scavenge reactive oxygen species (ROS) and lessen oxidative stress in the TME. According to Zhen et al., fullerene derivatives like β-alanine-modified gadofullerene (GF-Ala) polarized immunosuppressive M2-type macrophages towards the pro-inflammatory M1 phenotype, reducing expression of CD206, an M2-like tumor-associated macrophage marker, and increasing the expression of IL-12, CD86, and inducible nitric oxide synthase (iNOS) [135]. These effects led to the enhanced infiltration of cytotoxic CD8^+^ T cells and tumor regression in multiple murine cancer models, including 4T1 breast tumors and CT26 colon carcinoma [135]. Fullerenes’ surface chemistry also allows functionalization with polyethylene glycol (PEG) and loading with chemotherapy or checkpoint inhibitors for drug delivery applications. In “cold” tumor models, for example, a multifunctional nanotherapeutic system including GF-Ala, catalase, and the TLR7/8 agonist R848 in a PEG-PLGA matrix demonstrated significant TME remodeling, tumor regression, and inhibition of metastases [136]. Furthermore, fullerene nanomaterials have been shown to induce ICD and enhance dendritic cell maturation via redox-mediated tumor vascular disruption and photodynamic therapy (PDT) [136]. Ongoing innovations in surface modification, such as mannose or amino acid conjugation, are aimed at improving biosafety, targeting specificity, and translational feasibility in cancer immunotherapy.

#### 3.2.11. Magnetic Nanoparticles: Controlled Targeting and Imaging

The inherent magnetic responsiveness, biocompatibility, and multifunctionality of magnetic nanoparticles (MNPs), especially superparamagnetic iron oxide nanoparticles (SPIONs), make them a highly adaptable and clinically useful nanoplatform in cancer immunotherapy [137]. In terms of structure, SPIONs are made up of a core of either magnetite (FeO_4_) or maghemite (γ-FeO_3_), which are superparamagnetic at nanoscale sizes (<20 nm). This allows them to be magnetically steered to tumor locations without losing their magnetism after the external field is removed [137]. This magnetic targeting enables site-specific drug delivery, reducing off-target toxicity and enhancing therapeutic efficacy [138]. Additionally, SPIONs are great contrast agents for magnetic resonance imaging (MRI) due to their intrinsic magnetic characteristics, which enable the real-time, non-invasive monitoring of nanoparticle biodistribution and therapy response [139]. SPIONs have shown promise in immunotherapy in addition to imaging. When subjected to an alternating magnetic field, SPIONs can act as agents of magnetic hyperthermia by producing localized heat that causes ICD and encourages the release of pro-inflammatory cytokines and TAA [140]. In addition, SPIONs have been engineered for combination therapy, co-delivering chemotherapeutics and immunostimulants [141]. SPIONs can also be conjugated with fluorescent dyes and tumor-targeting ligands, enabling both MRI and optical imaging and improving diagnostic accuracy and treatment planning [142]. Clinically, SPION-enhanced MRI has demonstrated improved sensitivity in detecting micrometastases and assessing treatment response [137]. Challenges remain in optimizing stability, long-term biocompatibility, and clearance, and recent SPION formulations focus on improving surface coatings to minimize aggregation and enhance circulation time [143].

#### 3.2.12. Potential In Vivo Toxicities from Non-Lipid-Based Nanoparticle Treatments

Even though non-lipid-based nanoparticles have therapeutic potential in cancer immunotherapy, several formulations show possible in vivo toxicities that need to be addressed for safe clinical translation. For example, it is known that when QDs, especially cadmium-based core-shell structures like CdSe/ZnS, degrade, they emit harmful heavy metal ions like Cd^2+^, which can cause oxidative stress, mitochondrial dysfunction, and bioaccumulation in important organs [115]. This raises concerns for long-term biocompatibility, despite efforts to mitigate toxicity via PEGylation or silica coatings [118]. Similarly, when given systemically, CNTs pose hazards because of their poor biodegradability and the possibility of chronic inflammation and fibrotic reactions in pulmonary tissues. Studies on in vivo rodents have shown that exposure to CNTs causes granuloma formation and macrophage activation, emphasizing the need for surface modification to increase dispersibility and lower immunological activation [144]. Moreover, PACA nanoparticles, although biodegradable, have been shown to elicit acute immune responses and local cytotoxicity if not properly synthesized [145]. Even SPIONs, while clinically utilized for imaging, can induce dose-dependent cytotoxicity, particularly if they aggregate or persist in tissues [146]. These toxicities emphasize the importance of tailoring nanoparticle size, surface chemistry, and clearance kinetics to avoid adverse reactions while maximizing therapeutic index.

#### 3.2.13. FDA-Approved Treatments

The FDA in the United States has previously approved several polymers and nanoparticle compositions reviewed above, especially for use in cancer treatment. Because of their superior biocompatibility and regulated biodegradation into lactic and glycollic acid, poly(lactic-co-glycolic acid) (PLGA) is one of the most highly regulated and therapeutically used polymers. PLGA is notably utilized in Eligard^®^ (Tolmar Pharmacceuticals, Fort Collins, CO, USA) for endometriosis and Lupron Depot^®^ for prostate cancer. The FDA has also approved PEG as a surface modifier in Doxil^®^, a PEGylated liposomal doxorubicin that increases the drug’s solubility and circulation time [55]. Poly(lactic acid) (PLA) is another authorized system that is utilized in a variety of implantable devices and drug delivery devices [54]. Chitosan, though not yet FDA-approved as a drug carrier per se, is widely studied in investigational new drug (IND) applications and is still only approved as a food additive by the FDA [147]. Meanwhile, inorganic nanoparticles like SPIONs have been FDA-approved for clinical imaging purposes (e.g., Feridex^®^ (AMAG pharmaceuticals, Waltham, MA, USA) and Resovist^®^ (Bayer Pharmaceutical, Berlin, Germany)) and are under evaluation in immunotherapeutic contexts [148]. Other systems, such as PACA, carbon-based nanoparticles, and quantum dots, are actively advancing through preclinical and early-phase clinical studies, indicating their increasing translational potential, even if they have not yet received regulatory approval for therapeutic use.

#### 3.2.14. Lipid-Based Nanoparticle Delivery Systems

Non-lipid-based drug delivery methods and their uses in cancer immunotherapy have already been covered. In this section, we will review lipid-based nanoparticle delivery systems and their potential as a novel approach for cancer therapy.

#### 3.2.15. Liposomes

Liposomes are one of the most well-established nanoparticle systems in cancer therapy, with Doxil being the first liposomal drug delivery system approved by the Food and Drug Administration (FDA) in 1995 [149]. It is composed of phospholipids that encapsulate doxorubicin to reduce cardiotoxicity and improve drug accumulation at the tumor site via the EPR effect [150]. These liposomes are spherical vesicles made of a hydrophilic core and one or more phospholipid bilayers. This structure enables the encapsulation of both hydrophilic drugs in the liposomal core and hydrophobic drugs within the lipid bilayer [151]. However, the recognition of NPs by the reticuloendothelial system (RES) can hinder delivery into cells, resulting in liposomal clearance [152]. Various strategies have been established to reduce off-target effects and increase the liposomal stability and circulation time. PEGylated liposomes were one of the successful approaches utilized to evade the RES clearance and prolong systemic circulation [153]. This can be accomplished by covalently binding the PEG molecule to pre-formed liposomes or by directly adding polyethylene glycol (PEG)-conjugated phospholipids, like DSPE-PEG2000, to the lipid mixture during liposome synthesis. In comparison to non-PEGylated liposomes, a study by Hong et al. showed that PEGylated liposomes had longer circulation time because of decreased RES clearance [154]. However, the incorporation of PEG poses some challenges related to a hypersensitivity reaction known as complement activation-related pseudoallergy (CARPA), leading to significant immunogenicity [155]. Studies have demonstrated the ability of some molecules, such as Factor H (FH), in suppressing the complement activation induced by CARPA [156]. Liposomes have demonstrated significant promise in the field of immunotherapy for improving immunostimulatory drug distribution and optimizing therapeutic efficacy. PEGylated liposomes functionalized with interleukin-2 (IL-2) and anti-CD137, immunostimulatory agents that activate CTLs and NK cells, accumulated quickly towards the tumor, improved immune cell infiltration, and stimulated cytokine production [16]. These particles were able to penetrate the tumor microenvironment via the EPR effect and initiated a localized immune response. This approach showed significant antitumor effects in multiple tumor models, with reduced systemic toxicity compared to free drug [157,158]. A study conducted by Bayyurt et al. explored the delivery of dual TLR agonists, polyinosinic-polycytidylic acid; poly(I:C)—TLR3 and CpG oligodeoxynucleotides (ODN)—TLR9, by liposomes to activate immunity and prolong protection against tumors. The liposomal delivery system showed significant uptake of CPG and Poly(I:C) by the dendritic cells (DCs), with 2.5- and 5-fold increases, respectively, compared to unencapsulated ligands. When compared to the control group, the liposome formulation demonstrated a 75% tumor-free rate over 14 days and a significant reduction in tumor size [159]. In addition to surface functionalization, liposomes’ inherent properties can play a role in cell targeting. Positively charged liposomes have been shown to be able to target immune cells and stimulate an immunological response against tumors [160]. In a mouse model of colorectal cancer peritoneal metastases, a cationic DSTAP-liposome encapsulating resiquimod (R848), an aTLR7/8 agonist, produced a 4-fold increase in DCs, a 10-fold increase in CD4^+^ T cells, and a 5.2-fold increase in CD8^+^ T cells. When compared to free R848, DSTAP-liposome encapsulation greatly increased antitumor activity, with a 60% survival rate over 150 days [161]. The formulation was further improved by incorporating R8 peptide and anti-PD-1 immune checkpoint inhibitors (ICIs) to enhance cellular targeting and boost the survival rate to 90% in a carcinomatosis mouse model [162].

Since cancer cells are able to suppress the activation of immune cells through the inhibitory immune checkpoint pathway, efforts have been made to effectively deliver a blockade to the TME [163]. Notably, CTLA-4, PD-1, and PD-L1 are well-known inhibitory checkpoints and have been explored as an effective immunotherapy [164]. Ipilimumab is a monoclonal antibody against CTLA-4 that was approved by the FDA in 2011 for cancer treatment. However, adverse reactions due to immunogenicity have facilitated the use of liposomes to package and deliver ipilimumab into the targeted cells [165,166]. In a colon cancer mouse model, Nikpoor et al. developed anti-CTLA-4 liposomes, which showed enhanced tumor accumulation and a significant therapeutic effect when compared to free drugs [167]. Additionally, attempts have been made to investigate PD-1 pathways via liposomes. Anti-PD-1 antibody was added to liposomes by Zhou et al., who then investigated the therapeutic effect in a mouse model. Due to improved liposome uptake in the tumor microenvironment, the liposomal group showed a longer survival rate and smaller tumor size than the free anti-PD-1 group across three tumor models (H22, 4T1, and CT26), with statistically significant differences (*p* < 0.05) [168]. Additionally, Hei et al. used liposomes functionalized with anti-PD-L1 on the surface for increased targeting and reduce systemic toxicity [169]. Also, pH-sensitive liposomes were developed by Gu et al. that can selectively target tumor cells via anti-PD-L1 antibodies and further release docetaxel (DTX) intracellularly in response to the acidic TME [170]. Other immune checkpoints, such as LAG-3, TIM-3, and TIGIT, are under exploration as cancer immunotherapy and can possibly be integrated into liposomal delivery systems for more efficient cancer targeting [171,172,173].

Moreover, liposomes were investigated to encapsulate nucleic acids (e.g., mRNA, siRNA, DNA) to shield them from enzymatic degradation and effectively deliver them to the targeted cells [174]. Fusogenic liposomes show a promising approach in delivering nucleic acids to cells. Mimicking the behavior of extracellular vesicles (EVs), these liposomes consist of anionic lipids that are able to fuse directly with cellular membranes and release their cargo into the cytosol [175]. Stremersch et al. developed anionic fusogenic liposomes with cholesteryl hemisuccinate for siRNA delivery to the dendritic JAWSII cell line or H1299 non-small cell lung carcinoma. The in vitro study demonstrated significant downregulation of the targeted gene by the liposomal delivery of siRNA in both cell lines compared to the free form [176]. Lipoplex is another class of liposomes prepared by the electrostatic interaction achieved by the negatively charged nucleic acid and the pre-formulated cationic liposomes. Salomon et al. developed RNA-lipoplexes encoding CD4 neoantigen, demonstrating enhanced efficacy and remission in CT26 tumors in vivo [177]. Similarly, Kranz et al. investigated the efficacy of antigen-encoding RNA-lipoplexes in inducing an IFNα response on T cells as an immunotherapy approach. The group further investigated the effect of charge on the targeting efficiency and immune response through the variation of lipid:RNA ratios. Particle accumulation shifted to the spleen as a result of a gradual decrease in cationic lipid concentration, where slightly negatively charged particles showed increased RNA expression in splenic cells [178]. While these advances in liposomes provide a promising platform for immunotherapy, issues including the immunogenicity of PEGylated liposomes and off-target effects still need to be resolved (Table 2).

#### 3.2.16. Lipid Nanoparticles (LNPs)

With the success of the BNT162b2 and mRNA-127 COVID-19 vaccines, LNPs have drawn a lot of interest as a drug delivery platform (Table 2) [179]. They demonstrated efficient antigen expression and triggered a strong immune response. LNPs are generally composed of ionizable lipids, phospholipids, cholesterol, PEG-lipids, and the encapsulated nucleic acids [180,181]. They shield nucleic acid from enzymatic degradation and deliver it to the targeted cells. Upon cellular uptake, LNPs can promote endosomal escape, allowing their cargo to be released into the cytoplasm for a therapeutic effect [182]. The integration of ionizable lipids into LNPs enables complexation with the negatively charged nucleic acids during synthesis at acidic pH, where the ionizable lipids become protonated. At physiological pH, the ionizable lipids are neutral, resulting in longer circulation time in plasma. However, when LNPs enter the endosomes upon cellular uptake, the acidic environment (~5.5–6.0 pH) re-protonates the ionizable lipids, leading to membrane destabilization and cargo release in the cytosol [183]. The other components of LNPs, particularly phospholipids and cholesterol, provide structural integrity and stability. Additionally, PEG-lipids play a key role by initially increasing circulation time; however, the selection of the length and composition of the PEG chain dictates the appropriate time of dissociation from the particle surface [184]. Following systemic injection, PEG dissociation enables plasma protein adsorption on the LNP surface to engage with the targeted cell’s specific receptor, facilitating effective internalization through receptor–ligand interaction [185]. In cancer immunotherapy, LNPs can deliver siRNA or mRNA to reshape the TME by silencing immune checkpoints like PD-L1 or by inducing the expression of tumor-specific antigens. A study by Zhang et al. developed LNPs encapsulating mRNA encoding ovalbumin (OVA), a model antigen, to test the effect on the T-cell immune response. A library of ionizable lipids was established and tested with 12-carbon tail (12-C) lipid, demonstrating a significant mRNA translation and immune response resulting from an enhanced delivery to DCs. In addition to mRNA delivery and translation, 12-C lipid showed adjuvant properties through TLR-4 activation to also stimulate IL-12 cytokine production in DCs for antitumor efficacy [186]. Similarly, Liu et al. examined the intratumoral administration of LNP delivering IL-12 and IL-27 mRNAs composed of synthesized ionizable lipid with di-amino groups (DAL). In a mouse melanoma model, the delivery of LNPs promoted the infiltration of immune cells such as NK and T cells into the tumor area, resulting in an approximately 3-fold inhibition of B16F10 melanoma growth compared to the untreated group [187]. In addition, LNPs encapsulating mRNA encoding an anti-human epidermal growth factor receptor 2 (HER2) antibody, trastuzumab, were tested in a breast cancer model. The level of mRNA-translated trastuzumab antibody found in plasma was 64% higher compared to the injected trastuzumab over 30 days, even at a high dose of trastuzumab [188]. When tested in the HER2-positive model, mRNA-LNPs demonstrated significantly greater antitumor efficacy, with approximately a 4-fold reduction in tumor size compared to the HER2-negative mouse model [188]. Moreover, the co-delivery of PD-L siRNA-LNPs and target antigen mRNA in monocyte-derived cells significantly increased antigen-specific CD8+ T-cell responses by 4.5-fold compared to the control siRNA-LNP DC group after two weeks of stimulation. By silencing both PD-L1/PD-L2 immune checkpoints and activating antigen-specific pathways, this dual delivery approach demonstrated stimulatory potential in enhancing ex vivo responses in translated cancer patients [189]. Similarly, to boost mRNA-mediated immune responses for better cancer immunotherapy, Pam3 and OVA mRNAs co-delivered by LNPs stimulated TLR2/1 and TLR7/8 pathways, respectively [189]. Furthermore, LNPs are utilized to deliver the next generation of cancer immunotherapy by encapsulating Cas9 mRNA and sgRNAs (single-guide RNAs) for targeted gene editing. Rosenblum et al. developed CRISPR-LNPs against PLK1 to explore the potential of therapeutic genome editing into aggressive orthotopic glioblastoma. The results showed a 30% increase in survival over the control group and up to 70% in vivo gene editing in glioblastoma. The study examined EGFR-targeted LNPs that selectively delivered sgPLK1 to ovarian tumors, allowing for 80% gene editing and significant tumor suppression in addition to glioblastoma [190]. Currently, LNPs stand as the most efficient delivery system for targeted gene delivery, and with rapid advancements in this field, they hold significant promise for revolutionizing cancer immunotherapy.

#### 3.2.17. Solid Lipid Nanoparticles (SLNs) and Nanostructured Lipid Carriers (NLCs)

Solid lipids like triglycerides, fatty acids, or waxes make up SLNs, which are colloidal transporters that stay solid at body temperature and room temperature. Surfactants can stabilize their solid structural core, which lowers the interfacial tension between phases [191]. While SLNs are more effective than traditional liposomes at encapsulating hydrophobic drugs, they are susceptible to solid lipid crystallization when stored, leading to drug leakage and decreased encapsulation efficiency [191,192]. NLCs are the second generation of SLNs; they overcome the drug leakage limitation by combining solid and liquid lipids, inhibiting drug release upon storage [193]. As for cancer immunotherapy, Banerjee et al. developed SLNs (PSM) encapsulating paclitaxel and modified it with Tyr-3-octreotide, a specific ligand to somatostatin receptors (SSTRs), which are overexpressed on melanoma cells. These PSMs demonstrated a 6.6-fold increase in CD8+ T-cell infiltration and systemic immune response, resulting in a significant reduction in tumor volume [194]. Additionally, Wang et al. developed NLC co-delivering doxorubicin (Dox) and sorafenib (Sfn) for esophageal cancer therapy. NLCs were able to modulate the TME by reducing regulatory T cells (Tregs) and activating effector T cells, achieved by the delivery of Sfn. Subsequently, Dox induced immunogenic cell death, inhibited primary tumor growth, and generated an effective immune response, suppressing the growth of a newly implanted distant tumor [195]. Similar to other lipid-based particles, SLNs and NLCs are biocompatible, biodegradable, and can encapsulate hydrophobic and hydrophilic molecules; however, challenges such as physical instability, limited structural understanding, and scalability need to be addressed to optimize their pharmaceutical potential (Table 2) [196].

#### 3.2.18. Extracellular Vesicles (EVs)

EVs are membrane-bound particles released by cells into the extracellular space. They include several subtypes, such as exosomes and microvesicles, and are composed of phospholipid nanovesicles [197]. The EVs can carry a range of biomolecules, including proteins, lipids, and nucleic acids (mRNA, miRNA, DNA) to promote cellular communication and molecule transfer [198]. They offer personalized platforms for immune regulation along with customized therapeutic delivery, making them a potential strategy in immunotherapy. Specifically, tumor-derived EVs can decrease cytotoxic T-cell responses or modify Treg cells to limit immune cell activity [198]. A study conducted by Chen et al. identified circUSP7 exosomes as a crucial biomarker in non-small cell lung cancer (NSCLC). The study revealed the mechanism of circUSP7 in inhibiting the function of CD8+ T cells by downregulating the expression of miR-934 and subsequently reducing the therapeutic efficacy of anti-PD1 treatment [199]. Similarly, pancreatic cancer-derived EVs were transferred into T lymphocytes, inhibiting their antitumor capability. The derived EVs induce endoplasmic reticulum stress-mediated apoptosis in T lymphocytes through the activation of the p38 MAPK pathway [200]. In addition, the overexpression of intercellular adhesion molecule 1 (ICAM-1) and PD-L1 on melanoma-derived exosomes facilitates the binding to LFA-1 and PD-1 on T cells, respectively, mediating immune suppression [201]. Alternatively, EVs derived from antigen-presenting cells (APCs), such as DCs, carry tumor antigens, enabling the direct activation of T cells [202]. Recently, EVs have been engineered to deliver drugs and biomolecules for the precise modulation of immune or cancer cells. Lu et al. examined the ability of exosomes derived from DCs to activate the antigen-specific immune response. In a hepatocellular carcinoma (HCC) mouse model, the isolated EVs carrying α-fetoprotein (AFP), a liver protein highly expressed in HCC patients, demonstrated a strong antigen-specific response by shifting the TME from immuno-inhibitory to immunostimulatory post-administration. Specifically, EVs activated CD8+ cytotoxic T lymphocytes (CTLs) and enhanced natural killer (NK) cell activity, resulting in reduced tumor progression [203]. A study by Gunassekaran et al. developed an engineered M1 exosome to reprogram TAMs into M1-like macrophages. These exosomes are surface-modified with IL-4RPep-1 and encapsulated NF-κB p50 siRNA and miR-511-3p to specifically target IL-4R on M2 macrophages and suppress protumoral signaling, respectively. The results demonstrated the reprogramming of TAMs by increasing M1 pro-inflammatory cytokines and subsequently inhibiting tumor growth in vivo post-systemic administration compared to untargeted and control peptide-labeled exosomes [204]. Furthermore, CpG-STAT3ASO encapsulated in neural stem cell (NSC)-derived exosomes stimulated immune responses and demonstrated superior antitumor effects. The delivery of exosomes into TAM facilitates the silencing of the STAT3 pathway and activates glioma-associated myeloid cells, leading to tumor regression in mice [205]. In a mouse model of melanoma, Shin et al. examined the anticancer efficacy of EVs produced by IL-2-stimulated CD4+ T cells. When compared to unstimulated EVs, these EVs encapsulating miR-155-5p, miR-215-5p, and miR-375 showed increased CD8+ T-cell proliferation and significantly inhibited tumor growth by almost 1.5 times [206]. In a different study, the development of EVs from IL-2-functionalized transgenic T cells resulted in modifications to miRNA profiles, specifically those of miR-181a-3p and miR-223-3p. The IL2-sEVs promoted CD8+ T cell-mediated cytotoxicity in a melanoma model and effectively inhibited tumor growth and metastasis by a 4-fold reduction compared to the untreated control group [207]. Moreover, Li et al. developed exosomes carrying PD1 protein and the immune adjuvant imiquimod, displaying the efficient inhibition of PD1/PDL1 immune checkpoint and restored CD8+ T cell function in melanoma and breast cancer models [208]. Because of their biocompatibility, low immunogenicity, and capacity to spontaneously transport biomolecules between cells, EVs present unique advantages as a delivery mechanism in immunotherapy. But EVs are faced with a number of issues, such as scalability, heterogeneity, and precise targeting mechanisms (Table 2) [202]. Future advancement in exosome synthesis is needed to address these limitations and enhance personalized immunotherapy.

#### 3.2.19. Other Lipid-Based Systems

Despite their potential, other lipid-based systems that are used in immunotherapy are not as well studied. For example, biphasic dispersions called nanoemulsions (NEs) are made up of water, oil, and surfactants that interact to produce stable droplets [209,210]. These systems offer advantages such as improved solubility for hydrophobic drugs and can promote targeted drug delivery to immune cells. Although research on NEs in immunotherapy is still in its early stages, they have demonstrated potential in promoting tumor-specific immunity. Kuai et al. developed a multifunctional platform composed of perfluorooctyl bromide (PFOB) nanoemulsions holding MnO_2_ nanoparticles (MBP) for tumor-targeted immunotherapy and dual-modality imaging (MRI/CT). The findings showed that MBP nanoemulsions successfully prevented tumor development and lung metastasis by inducing immunogenic cell death (ICD) and CD8+/CD4+ T-cell activation [211]. Another study by Zeng et al. developed an oil-in-water nanoemulsion that encapsulated the OVA antigen within the oil core and targeted the C-type lectin receptor, Clec9A, which is expressed on DCs. In aggressive models of breast cancer and B16-F10 melanoma, this strategy significantly reduced tumor growth and produced antigen-specific CD8+ and CD4+ T-cell responses [212].

One promising delivery method for cancer immunotherapy is Lipid Polymer Hybrid Nanoparticles (LPHNPs). They are a powerful nanocarrier system that combines the unique characteristics of polymers and lipids [213]. These nanoparticles usually include a lipid shell enclosing a polymeric core, which offers controlled release profiles, effective drug encapsulation, and structural stability. The polymeric core allows for precise drug loading and regulated release, while the lipid shell improves biocompatibility and decreases opsonization [214]. In immunotherapy, LPHNPs have gained attention for their ability to co-deliver multiple therapeutic agents. They are able to encapsulate hydrophilic and hydrophobic molecules, including mRNA, DNA, and small-molecule drugs [215]. According to recent studies, they can boost checkpoint inhibitor delivery effectiveness and stimulate antitumoral immune activity. Tin mesoporphyrin (SnMP), an inhibitor of Heme oxygenase 1 (HO1), was added to LPHNPs by Yong et al. to target bone marrow myeloid and leukemic cells. In a human AML-bearing mouse model, the particles showed increased inflammatory gene expression and immunological responses [216]. While these systems have shown great potential in boosting immunotherapy, additional research and thorough investigations are required to maximize their effectiveness, safety, and clinical suitability.

**Scheme 1:**
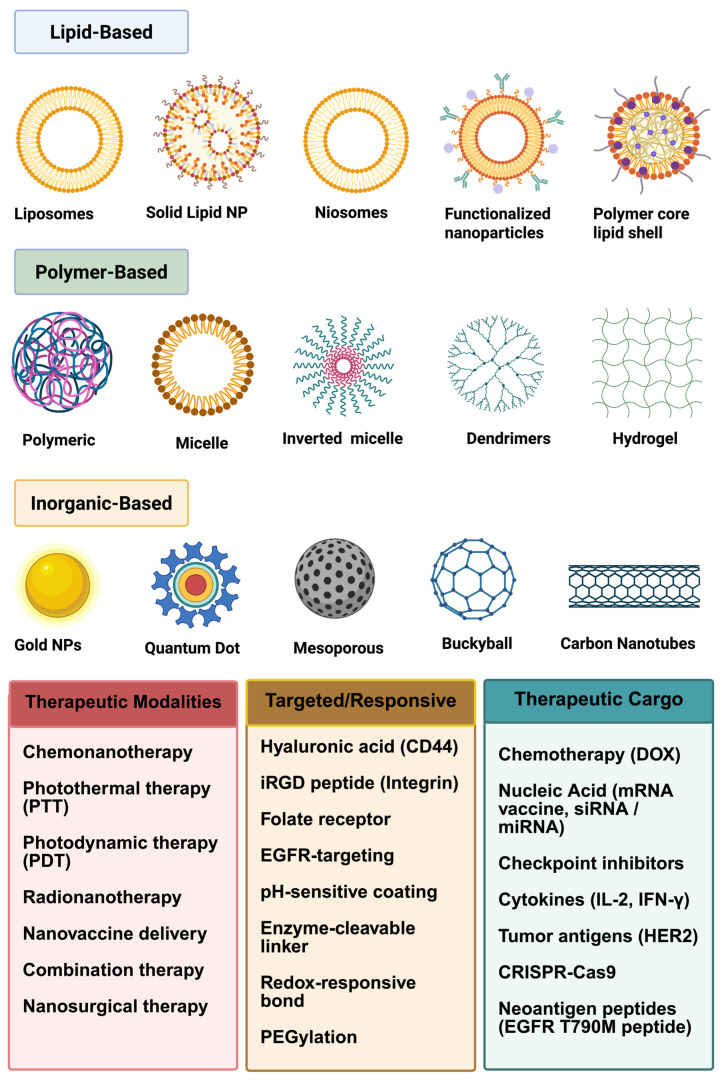
Overview of representative nanocarrier systems, therapeutic strategies, targeting mechanisms, and bioactive cargos used in cancer immunotherapy. Nanoparticles are classified by composition into lipid-based (e.g., liposomes, solid lipid nanoparticles, niosomes), polymer-based (e.g., polymeric nanoparticles, micelles, dendrimers, hydrogels), and inorganic-based systems (e.g., gold nanoparticles, quantum dots, mesoporous silica, carbon allotropes). Key therapeutic modalities include chemonanotherapy, photothermal and photodynamic therapy, and nanovaccine delivery. Functional surface modifications such as hyaluronic acid (CD44-targeting) and iRGD peptides facilitate tumor-specific delivery, while therapeutic cargos span small-molecule chemotherapeutics (e.g., doxorubicin), nucleic acids (mRNA, siRNA, miRNA), immune checkpoint inhibitors, cytokines (IL-2, IFN-γ), tumor-associated antigens (e.g., HER2), genome-editing tools (CRISPR-Cas9), and neoantigen peptides (e.g., EGFR T790M).

Collectively, these lipid-based drug delivery methods have become revolutionary cancer immunotherapy platforms. These systems can regulate immune responses, improve drug stability, and enable targeted administration. They also deal with issues including immunosuppression in the TME and off-target effects. Despite their progress, limitations such as scalability and immune-related side effects remain challenging (Table 2). Future advancements in engineering these platforms can improve therapeutic efficacy and enhance cancer immunotherapy. Scheme 1 illustrates different lipid, polymer, and inorganic-based nanocarriers and summarizes their therapeutic strategies, targeting mechanisms, and their bioactive cargo used in cancer immunotherapy.

### 3.3. Protein-Based Nanoparticles

Protein-based nanoparticles are natural molecules that have emerged as potential drug delivery approaches in the cancer immunotherapy field with multiple functionalities [217,218]. This nanodrug delivery approach can be synthesized from subunits of a particular protein or a group of different proteins, including water-soluble and insoluble proteins, providing numerous moieties that can be utilized to generate binding sites for the drug or imaging molecules, making them suitable for theranostics in the treatment of diseases [217]. Because protein-based nanoparticles are composed of natural proteins, they have outstanding biocompatibility, biodegradability, and high drug loading capacity, and they are easily amenable to functional modifications to facilitate drug and target antigen binding. Plus, protein-based nanoparticles can control the distribution of the nanodrug within the tissue [219]. Some of the proteins that have been used for protein-based nanoparticle formulation include albumin, gelatin, Elastin, zein, Gliadin and Legumin, soy proteins, milk proteins, and whey proteins [218]. Due to their nature and remarkable characteristics, this drug delivery approach has had one of the greatest successes in the clinic for cancer treatment [220]. For example, Abraxane, which is a form of protein-based nanoparticle conjugated with the chemotherapy drug paclitaxel (PTX), has been used in the clinic to treat different tumor models, including pancreatic cancer, non-small cell lung cancer, and breast cancer, owing to the biocompatibility and targeted ability of the drug [219]. Ontak (Eisai) is another protein-based drug combined with L-2 and diphtheria toxin used to treat T-cell lymphoma. This drug combines a lysosomal escape property and the ability to specifically bind to T cells, providing precise and more effective treatment efficacy [219]. Although protein-based nanoparticles have shown promising, encouraging results in the clinic, further investigation into their performance and treatment efficiency is still required.

## 4. Applications in Cancer Immunotherapy

Through immune response enhancement and TME modulation, gene delivery methods have significantly contributed to the improvement of cancer immunotherapy. To improve immune cell function, this can be accomplished by effectively transferring genetic materials to cancerous or immune cells [221]. For example, delivering cytokine genes such as IL-2 enhances immune infiltration in the tumor microenvironment [222]. Similarly, delivering genes that encode tumor antigens increases the immune system’s ability to recognize cancer cells [223].

Gene-regulating nucleic acid can modulate gene expression by either upregulating or downregulating specific genes to allow the targeting of genetic pathways involved in diseases [224]. They provide an appealing treatment option for diseases like cancer brought on by dysregulated or mutant genes. Single-stranded DNA or RNA molecules known as antisense oligonucleotides (ASOs) attach to specific mRNA sequences to prevent translation [225]. As an immunotherapy approach, an ASO was synthesized to target CD39 mRNA, an ectonucleotidase that converts extracellular ATP into immunosuppressive adenosine to knock down its expression in a colorectal adenocarcinoma mouse model. The results show enhanced T-cell proliferation and improved immune responses against tumor cells [226]. Similarly, siRNA is able to silence immune checkpoint proteins in tumor cells to restore the activity of T cells by targeting a specific mRNA sequence [227]. Warashina et al. developed a novel LNP capable of delivering siRNA efficiently to DCs. The delivered siRNA demonstrated over 80% silencing of the *SOCS1* gene, resulting in cytokine production and inhibition of tumor growth in a lymphoma mouse model [228]. Furthermore, Song et al. utilized pH-sensitive polymeric nanoparticles for the co-delivery of *VEGF* and *PIGF* siRNA to TAM and cancer cells. The study revealed the inhibition of breast cancer proliferation and reprogramming of the tumor microenvironment toward a more immunogenic state. Once internalized in M2-TAMs and tumor cells, the NPs disassemble in response to the acidic environment, releasing siRNA into the cytoplasm, which subsequently silences the *VEGF* and *PIGF* genes [229].

Small non-coding RNAs known as microRNAs (miRNAs) are becoming important modulators in cancer treatment [230,231]. Various delivery systems have been utilized to deliver miRNA to maximize its therapeutic potential. For instance, Chen et al. formulated a system of liposome–polycation–hyaluronic acid nanoparticle for the delivery of siRNAs and miRNA into murine B16F10 melanoma. The study revealed tumor regression through the synergistic action of siRNAs and miR-34a by silencing the *c-Myc*, *MDM2*, and *VEGF* genes and activating the p53 pathway, leading to tumor apoptosis [222]. Additionally, PLGA-based nanoparticles demonstrated the successful delivery of miR-150 to pancreatic cancer cells. The MUC4 gene, which promotes tumor progression in pancreatic tumors, was downregulated by miR-150 administration, significantly suppressing the growth of tumor cells [232]. Devulapally et al. showed that PEGylated-PLGA NPs containing gemcitabine (GEM) and antisense-miRNA-21 could successfully stop the growth of hepatocellular carcinoma (HCC) cells and cause cytotoxicity. The combination therapy resulted in the downregulation of miRNA-21, restoring tumor suppressor expression and releasing GEM in HCC cells, effectively inducing apoptosis [233]. Moreover, a study conducted by Parayath et al. revealed that hyaluronic acid-poly(ethylenimine) NPs effectively target CD44 and deliver encapsulated miR-125b to macrophages in lung tissues. The delivery of miR-125b to peritoneal macrophages re-polarized M2 into M1 macrophages for an antitumoral and pro-inflammatory response in non-small cell lung cancer (NSCLC) [234].

mRNA-based cancer vaccines are a revolutionary approach to immunotherapy and gene delivery. These vaccines activate the immune system to identify and eradicate cancer cells by encoding neoantigens or tumor-specific antigens [235]. Following vaccination, antigen-presenting cells (APCs), including DCs, receive the mRNA, which is then converted into tumor-specific proteins (antigens). Following the cell’s processing of these antigens, major histocompatibility complex (MHC) molecules present them on the cell surface, triggering an immune response [236]. The mRNA cancer vaccine can induce both cellular and/or humoral immunity to create a long-lasting immune memory. The successful emergence of LNPs as a non-viral gene delivery system advanced the delivery of mRNA vaccines into targeted cells [237]. Various studies have revealed the mRNA-mediated delivery of antibodies to activate antitumor immune responses. For example, Thran et al. showed that mRNA-LNP encoded for rituximab, a CD20-targeting monoclonal antibody, and were able to enhance antitumor efficacy compared to traditional monoclonal antibody therapy. The mRNA-LNP treatment enhanced mouse survival by approximately 64.7%, with the control group euthanized by day 17, while the mRNA-LNP group survived until the end of the study of 28 days [238]. In a different study, mRNA-LNPs encoding M1 and atezolizumab, anti-PD-L1 antibodies, demonstrated reduced tumor growth in a colon adenocarcinoma mouse model [239]. The utilization of mRNA encoding a toxic protein is another approach for mRNA cancer vaccines. Jian et al. developed a novel approach targeting HCC using mRNA-LNPs encoding apoptotic proteins (e.g., Caspase or PUMA). The synthesized mRNA incorporated complementary microRNA target sites able to suppress the translation of the apoptotic mRNA in healthy cells. For example, miR122 is selectively expressed in healthy hepatocytes but not in HCC cells. This approach can effectively induce apoptosis in tumor cells while minimizing off-target effects [240]. Furthermore, mRNA could modify the TME. In a mouse melanoma model, the intratumoral delivery of LNPs encapsulating IL-12 and IL-27 mRNAs showed sustained tumor growth suppression. NK and CD8+ T lymphocytes were successfully infiltrated into the TME by the translation of the supplied mRNAs [187]. In their study, Bevers et al. developed mRNA-LNP vaccines encoding the HPV16 E7 antigen and combined with TriMix (mRNAs for CD70, CD40L, and caTLR4). The results demonstrated significant infiltration of CD8+ T cells and tumor regression in TC-1 tumor-bearing mice [241]. Overall, mRNA vaccines represent a transformative approach in immunotherapy, offering flexibility and precision. However, challenges such as enhancing delivery efficiency to target cells and addressing manufacturing scalability must be addressed to optimize their therapeutic potential.

Currently, CRISPR-based gene editing represents a significant advancement in cancer immunotherapy. This technology makes it possible to precisely modify immune cells, such as by deleting inhibitory genes or causing tumor cells to produce immunogenic neoantigens [242]. Luo et al. developed cationic lipid-assisted PEG-b-PLGA nanoparticles (CLANs) that included guide RNA directed at the Ntn1 gene, which controls inflammation and macrophage activity, as well as promoter-driven Cas9 plasmids (pM330) unique to macrophages. When CRISPR/Cas9 is successfully delivered, it precisely impacts the Ntn1 gene in macrophages, which lowers netrin-1 production and restores the balance of immune cells [243]. Another study developed pH-responsive nanoparticles co-loaded with paclitaxel and CRISPR/Cas9 to target the *Cdk5* gene. These particles are composed of a cationic PEI−PLGA core and an acid-cleavable PEG corona. Upon delivery to the tumor cells, the released Cas9-Cdk5 knocks out the *Cdk5* gene and reduces PD-L1 expression on tumor cells to restore cytotoxic T-cell activity. Simultaneously, the delivered PTX induces immunogenic cell death, creating a synergistic immunochemotherapy effect [244]. In addition, Cheng et al. produced human serum albumin (HSA) NPs by combining non-covalently bound CRISPR/Cas9 or siRNA with a double emulsion of stearyl-polyethylenimine (stPEI)-complexed plasmids. T cell-mediated immune responses were reactivated, and PD-L1 expression in tumor cells was silenced as a result of the study’s 21.95% decrease in gene knockdown [245]. Arginine-coated gold nanoparticles (ArgNPs) are another strategy that can be used to deliver CRISPR-Cas9 to cells. Ray et al. developed ArgNPs encapsulating CRISPR-Cas9 to knockout the *SIRP-α* gene in macrophages responsible for interacting with CD47, a cell surface protein that functions as a “don’t eat me” signal that inhibits macrophages from phagocytosing cancer cells. This system achieved 90% delivery and 30% gene-editing efficiency in vitro, which enabled cancer cell phagocytosis, making it a promising approach for cancer immunotherapy [246]. Moreover, Leonard et al. investigated the use of CRISPR-RICTOR-liposomes to modify and control macrophage polarization in breast cancer liver metastases (BCLM). The delivered CRISPR-Cas9 could target the *RICTOR* gene responsible for driving macrophage differentiation towards the M2 phenotype. The results revealed a preference for polarization towards the pro-inflammatory M1 phenotype, which supports tumor regression [247]. Despite the CRISPR editing approach holding promise for advancing immunotherapy and enhancing immune cell function, challenges remain. These include achieving efficient and specific delivery to target cells and minimizing off-target effects. Addressing these hurdles is essential to fully realize its therapeutic potential and translate these innovations into safe and effective clinical applications [248].

## 5. Biocompatibility and Safety Concerns

Nanoparticle design and manufacturing requires careful attention to specific details, including the initial selection of the materials, the production methods, and product purification at the final stages of manufacturing [249,250]. Although the safety profiles of commonly used materials, such as phospholipids and biodegradable polymers, are well documented, nanoparticles become more complex with the inclusion of various synthetic compositions [249,251]. For example, with surface coatings and ligands, their biocompatibility, biodistribution, and overall toxicological profiles may change. Thus, rigorous evaluation is necessary to get the most optimal characteristics [249,251].

Despite their promising physicochemical and biological properties, nanoparticles as drug delivery tools and therapeutic agents have their own considerable challenges due to their particulate nature. These include the possibility of experiencing adverse immune events such as allergies or hypersensitivity [249,251]. Therefore, it is crucial to take these challenges into account to minimize undesirable side effects. Additionally, there is a significant discrepancy between the success of nanotherapeutics in preclinical studies and their performance in clinical trials. This is partly due to the limitations of current animal models that are used for nanoparticle studies that do not mimic the complexity and heterogeneity of human cancer, including tumor metastasis, which is a major contributor to cancer mortality [249,251]. Improvements in the translation of therapeutic nanoparticles could be achieved by developing new animal models that can mimic the unique attributes of human tumors [249]. Table 3 presents a summary of important challenges of different cancer nanoparticle formulations.

As described earlier, nanoparticles can be utilized to deliver various therapeutic agents to kill tumors. However, non-PEGylated liposomes, such as those containing doxorubicin, tend to accumulate in non-target organs like the liver, lungs, and spleen due to their high affinity for the reticuloendothelial system [252,253]. This accumulation is primarily driven by opsonization, which facilitates their rapid removal from blood circulation. In contrast, PEGylated liposomes are designed to minimize their affinity to RES, significantly reducing macrophage uptake and potentially enhancing therapeutic targeting [253].

One of the most critical observations that have been reported for using nanoparticles is the initiation of complement activation-related pseudoallergy (CARPA), a non-IgE-mediated hypersensitivity reaction that can result in severe symptoms, including anaphylaxis [254,255]. Managing such reactions typically involves the modification of the drug administration plan or the use of standard allergy medications to overcome the side effects [255]. Additionally, the development of immunogenic responses to nanoparticulate-based therapies could significantly alter the pharmacokinetics of the drug, leading to serious toxicities [254].

Some clinical trials that involve nanomaterials fail due to nanotoxicity issues, where nanoplatforms alter drug biodistribution, causing drugs to accumulate in organs like the liver, lungs, or spleen to a greater extent than when they are administered in free molecular form [251]. For instance, the phase 1 clinical trial of the liposomal drug MRX34 was halted due to serious immune-related side effects [251]. Addressing safety concerns related to nanodrug biodistribution and enhancing therapeutic performance are critical goals for more successful clinical applications of nanomedicines. Moreover, the biocompatibility and biodegradability of nanoparticles must be carefully considered. Current safety evaluation methods for nanoparticle use are similar to those used for traditional drugs, emphasizing the urgent need for new approaches to detect nanotoxicity. Optimizing the size and surface characteristics of nanoparticles can improve their biocompatibility and biodistribution, enabling them to navigate various tissue barriers more effectively [251].

The clinical translation of extracellular vesicle (EV)-based cancer therapies results in a significant need for safety, given EVs’ potential capability as highly biocompatible delivery vehicles with minimal immunogenicity [256]. Numerous preclinical and clinical studies have generally supported the safety of EVs, highlighting their low toxicity and lower immunogenic responses [256,257]. For instance, studies have shown that high doses of EVs do not cause structural or functional changes in hepatoblastoma cells (HepG2) and do not trigger inflammatory responses, nor do they induce toxicity or severe immune reactions in immune-competent mice [256]. Furthermore, EVs derived from tumor cells were found not to affect oncogenic or DNA damage pathways in these cells [256]. Despite the promising safety profiles observed, further detailed studies are needed to fully understand the implications of using EVs as a potential therapy for cancer. Key areas for future research include the effects of repeated EV injections, different administration routes, and the use of EVs from various cell types. Such investigations are essential to ensure the safety and efficacy of EV-based therapies prior to their broader clinical application [256].

## 6. Regulations and Manufacturing Barriers

Regulations and scientific challenges significantly impact the development and approval of nanoparticles [254,258]. The current regulatory frameworks that are primarily designed for traditional medicine often fall short in addressing the unique complexities that are associated with nanoparticles [254,258]. For example, their intricate structures, interactions with biological systems, and their multifunctional properties that combine therapeutic and diagnostic capabilities [254]. Until recently, the FDA treated nanomedicines on a case-by-case basis, without established guidelines due to their complex nature, and categorized them neither as universally safe nor harmful. However, growing concerns about nanomedicine-associated risks, including toxicity and long-term effects, have prompted the FDA to issue one draft and five final guidance documents aimed at aiding manufacturers through the regulatory processes [253].

On the other hand, the European Medicines Agency (EMA) classifies nanomedicine as either biological or nonbiological, requiring extensive studies to ensure bioequivalence, safety, quality, and efficacy, which are essential for establishing therapeutic equivalence [253,258]. The regulatory landscape for nanomedicine involves intricate evaluations due to their distinct physicochemical properties and clinical behaviors, presenting particular challenges for generic nanomedicines in proving bioequivalence to their branded counterparts [253]. Additionally, nanomedicine development demands rigorous early-stage characterization and scalable manufacturing processes to guarantee reproducibility, quality, and effectiveness [253]. Although efforts provided by both the FDA and the EMA to provide structured guidance, the continually evolving nature of nanotechnology poses ongoing challenges to existing regulatory frameworks [253].

The lack of specific regulatory guidelines for nanomedicines in general has presented significant challenges in their production processes, unveiling considerable gaps in quality and safety information [259]. These limitations are often linked to high failure rates in clinical trials. In response, the EMA and the FDA have adopted a cautious approach by evaluating nanoparticle products under the umbrella of existing comprehensive regulations [259]. Nevertheless, there is an increasing need for more specific guidelines that directly address the unique challenges posed by nanomedicines. Currently, new initiatives are under development to set the guidelines, pressing the importance of proactive regulatory participation from the early stages of nanomedicine development [259]. Regulatory hurdles further complicate the scaling process due to the absence of standardized evaluation methods for nanomedicines, which are uniquely categorized under regulatory frameworks like the Public Health Service Act (PHSA) [253]. Additionally, there is an increasing pressing need for developing new regulatory standards and protocols specifically tailored for nanomedicines, taking into account their complex nature and specific characteristics, such as their pharmacokinetics and pharmacodynamics [254]. These factors underscore the need for careful planning and advanced methodologies in the production and approval of nanoparticle-based therapies [253]. Despite the potential benefits of nanoparticles, their development is often hindered by issues related to chemistry, manufacturing, and controls (nano-CMC) [250]. Critical early attention is required for the use of high-grade and affordable raw materials, proper segregation of materials and products in industrial facilities, avoidance of lengthy and low-yield synthetic steps, and simplification of overly complex designs [250]. Other challenges include the removal of difficult catalysts or impurities, variability caused by sensitive synthetic parameters, and the adoption of closed systems or automation to reduce costs and minimize errors [250]. Moreover, stringent general and nano-specific quality controls; thorough risk assessments for nano safety, stability, and shelf-life; and monitoring for endotoxin contamination and the unique properties of nanomaterials are essential [250]. Maintaining cost-effectiveness remains a fundamental requirement throughout the development process [250].

To make continued progress in the field of nanoparticles as promising tools for cancer therapy, it is essential to develop global regulatory standards, which requires collaborative work between international regulatory agencies, academia, and industry [142,145]. This step is crucial due to the limited number of specialized contract manufacturing organizations that are capable of producing nanoparticles in accordance with Good Manufacturing Practice (GMP). Additionally, a comprehensive evaluation and the documentation of nanoparticle production processes are necessary to meet industrial standards for quality control and environmental safety [254]. The development of new analytical tools and standardized methods is also critical for an accurate assessment of critical physical characteristics of NNMs, such as particle size, surface chemistry, and morphology, which significantly influence their performance in vivo [254].

Scaling up pharmaceutical nanoparticles presents several complex challenges. The intricate nature of these nanoparticles requires a comprehensive understanding to identify key characteristics that are essential for reproducibility [253]. This step is crucial for selecting suitable large-scale manufacturing processes and defining critical analytical criteria. Plus, sterilization methods such as gamma irradiation or autoclaving pose some risks, especially for nanoparticles containing biological materials, as they can damage them [253]. For instance, PLGA nano-formulations offer significant therapeutic benefits, but they face some challenges when it comes to large-scale production, such as batch-to-batch variations and inconsistencies during scale-up [260]. However, these challenges can be mitigated by using continuous process technologies. Due to the complexity of these formulations, collaboration among interdisciplinary experts is crucial for effective data management throughout the design processes. To leverage the unique capabilities of various stakeholders, fostering cooperation among large pharmaceutical companies, biotech firms, small enterprises, and academia through integrated consortia is essential. It is also expected that biopharmaceutical companies will increasingly outsource to Contract Manufacturing Organizations (CMOs) and Contract Research Organizations (CROs) to utilize their specialized expertise in the field [260].

The transition from basic research to the commercial production of therapeutic nanoparticles requires addressing several Chemistry, Manufacturing, and Control (CMC)-related challenges. A critical aspect of this process is demonstrating the ability to transfer a particular technology to a development facility or to a Contract Manufacturing Organization, where large-scale, cost-effective, and well-controlled production processes can be established under Good Laboratory Practice (GLP) and, ultimately, Good Manufacturing Practice (GMP) conditions [250]. Moreover, the transition from preclinical to clinical development and commercialization of therapeutic nanoparticles is challenging due to the complexity of their manufacturing [249]. To achieve the required standards for GMP that are necessary for the transition, it is suggested to utilize existing manufacturing unit operations within the pharmaceutical industry [249].

These issues collectively highlight the range of technical and regulatory challenges in the development of nanoparticles for cancer immunotherapy. Major hurdles include the need for GMP-compliant large-scale production, detailed quality control, specialized toxicology studies, and a deep understanding of nanoparticle interactions with biological systems. Additionally, challenges such as ensuring structural stability post-administration, overcoming limited drug accumulation in targeted areas, navigating the complex patent landscape, and understanding the biological interactions within the body should be considered for the success of nanoparticle drug manufacturing in the field of cancer immunotherapy [254].

## 7. Future Perspectives

The field of drug delivery in cancer immunotherapy is evolving rapidly with the integration of novel interdisciplinary strategies, technological advancements, and improved biomaterial designs. Therefore, several future directions could significantly enhance treatment outcomes.

The utilization of nanoparticles in cancer immunotherapy might be a promising approach to enhance the activation of the immune system for more effective anticancer immune responses to kill tumors. Particularly, engineered nanoparticles can be used as a carrier to deliver immunomodulatory molecules, enabling the precise delivery of tumor antigens to antigen-presenting cells (APCs) and lymphocytes [261]. It has been shown that the delivery of tumor-associated antigens (TAAs), adjuvants, or immune checkpoint inhibitors by engineered NPs can enhance the immune response and help overcome cancer progression [262]. Moreover, combining CRISPR/Cas9 with variable delivery systems could help in reprogramming immune cells, such as T cells and NK cells, by editing their genomes to enhance tumor cell recognition and eradication. In addition, gene editing can effectively change the behavior of the immune cells, enabling them to resist immunosuppressive signals and mount a more effective anticancer response [263].

Personalized medicine is progressively influencing the future of cancer treatments, highlighting patient-specific requirements for cancer treatment and their response to a particular drug. The advancement in omics sciences (e.g., genomics, proteomics) allows for the identification of unique biomarkers in tumors, which facilitates the development of personalized medicine. Furthermore, studies emphasizing the capability of bioengineered nanoparticles containing neoantigen vaccines to provoke strong immune responses will continue to grow; these types of vaccines are designed for a specific tumor characteristic, thus improving drug specificity and accelerating tumor eradication by the immune cells [264,265]. In addition, circulating tumor DNA (ctDNA) analysis could facilitate the real-time monitoring of treatment responses and guide adaptive therapy strategies by revealing genetic mutations or changes in the tumor burden. This genomic information allows clinicians to tailor more effective treatment plans. For example, by identifying resistance mutations early, switching to more effective anticancer therapies, or adjusting drug dosages based on tumor evolution over time [266].

Furthermore, artificial intelligence (AI) and machine learning (ML) are increasingly integral to enhancing nanomedicine design, formulation, and the prediction of therapeutic efficacy [267]. AI-driven computational models may swiftly assess extensive datasets to predict nanoparticle behavior, enhance drug loading efficiency, and customize nanoparticle surface characteristics for improved tumor targeting [267]. Research has demonstrated that AI-based algorithms can identify optimal nanoparticle compositions for improved pharmacokinetics and biodistribution, reducing systemic toxicity while maximizing therapeutic accumulation within the TME [268,269]. Furthermore, utilizing AI-assisted tools for imaging and diagnostics can aid in the real-time monitoring of the administered drug and its treatment efficacy [268].

Multifunctional characteristics of the next generation of drug delivery devices will allow simultaneous therapy and diagnosis (theranostics). Smart nanocarriers can respond to tumor-specific cues, such as pH, redox gradients, hypoxia, or enzyme activity, to achieve on-demand drug release, enhanced tumor penetration, reduced systemic toxicity, and improved therapeutic efficacy by selectively targeting cancer cells while sparing healthy tissues [270]. Hybrid nanoparticles, such as liposome–polymer conjugates and metal–organic frameworks (MOFs), have demonstrated enhanced drug loading capacity with controlled and stimuli-responsive drug release. Additionally, MOFs improved biocompatibility and showed synergistic therapeutic effects in cancer treatment by enabling the co-delivery of multiple therapeutic agents with distinct mechanisms of action [271,272]. Thus, MOFs might have a great impact on improving cancer therapy in the future.

## Figures and Tables

**Table 2:** Advantages and limitations of lipid-based nanoparticle delivery systems.

Delivery System	Advantages	Limitations
Liposomes	- Biocompatible and biodegradable. - Capable of encapsulating hydrophilic and hydrophobic drugs. - Low immunogenicity and customizable targeting options.	- Poor stability issues, drug leakage. - Limited scalability for large-scale production. - Short half-life in circulation without modification. - Problematic immunocompatibility.
Lipid Nanoparticles (LNPs)	- Biocompatible and biodegradable. - High encapsulation efficiency for mRNA and siRNA. - Reduced systemic toxicity. - Scalable production. - Proven clinical success in mRNA vaccines (e.g., COVID-19).	- Potential for inflammatory side effects due to ionizable lipids. - Limited specificity without additional targeting ligands.
Extracellular Vesicles (EVs)	- Naturally derived, biocompatible, and low immunogenicity. - Intrinsic targeting properties to specific cells or tissues. - Ability to carry a wide variety of biomolecules (e.g., proteins, RNA).	- Complex isolation and purification processes. - Limited scalability for mass production. - Heterogeneous physicochemical properties.
Solid Lipid Nanoparticles (SLNs)	- Stable in solid form and suitable for hydrophobic drug delivery. - Biodegradable and approved lipid components. - Scalable production through high-pressure homogenization.	- Crystallization can lead to drug expulsion. - Limited loading capacity for certain drug types.
Nanostructured Lipid Carriers (NLCs)	- Enhanced drug loading capacity compared to SLNs. - Improved physical stability over SLNs. - Effective for hydrophobic and poorly soluble drugs.	- Potential for particle aggregation during storage. - Complexity in formulation design.
Nanoemulsions	- High bioavailability and capacity for hydrophobic drug delivery. - Scalable and cost-effective production methods. - Versatile for multimodal imaging and immunotherapy.	- Requires energy-intensive processes (e.g., high-pressure homogenization) for formulation. - Poor drug-loading efficiency. - Toxicity of emulsifier.
Lipid–Polymer Hybrid Nanoparticles (LPHNPs)	- Combines the benefits of lipids and polymers for enhanced stability and drug release control. - Targeted delivery capabilities with dual cell targeting potential. - Ability to carry a wide variety of therapeutics (e.g., proteins, RNA).	- Complexity in synthesis and optimization of polymer–lipid ratios. - Potential cytotoxicity from certain polymer components. - Difficult to scale up and high production costs.

**Table 3:** Examples of challenges of nanoparticles in cancer immunotherapy [251].

Cancer Nanoparticles	Challenges
Liposomes	- Challenges in the production of liposomes - Elevated scale-up costs, inadequate therapeutic effectiveness - Inadequate clinical targeting - Tend to accumulate in reticuloendothelial system (RES)
Polymeric micelles	- Unstable in blood circulation - Storage challenges - Inadequate therapeutic effectiveness - Inadequate clinical targeting
Nano-biomaterials	- Inadequate therapeutic effectiveness - Complex and expensive scale-up manufacturing - Tend to accumulate in specific body systems - Storage challenges - Inconsistent repeatability

## Data Availability

Not applicable.

## Abbreviations

AuNPs	Gold Nanoparticles
AFP	Alpha-Fetoprotein
AML	Acute Myeloid Leukemia
APCs	Antigen-Presenting Cells
ArgNPs	Arginine-coated Gold Nanoparticles
ASO	Antisense Oligonucleotide
A-MnO_2_ NPs	Albumin -Manganese oxide Nanoparticles
A549	Lung carcinoma epithelial cells
AI	Artificial intelligence
AgNPs	Silver nanoparticles
BBB	Blood–Brain Barrier
BCLM	Breast Cancer Liver Metastases
BCRP	Breast Cancer Resistance Protein
Bcl-2	B-cell lymphoma 2
CD	Cluster of Differentiation (e.g., CD8^+^ T cells, CD86)
CNTs	Carbon Nanotubes
CARPA	Complement Activation-Related Pseudoallergy
Cdk5	Cyclin-Dependent Kinase 5
CT	Computed Tomography
CTLA-4	Cytotoxic T-Lymphocyte-Associated Protein 4
CTLs	Cytotoxic T Lymphocytes
CD73	Cluster of Differentiation 73
COX-2	Cyclooxygenase-2
CD206	Cluster of Differentiation 206
CRISPR/Cas9	Clustered Regularly Interspaced Short Palindromic Repeats and CRISPR-associated protein 9
ctDNA	Circulating tumor DNA
CMC	chemistry, manufacturing, and controls
CMO	Contract Manufacturing Organization
CRO	Contract Research Organization
CAR	chimeric antigen receptor
DAMPs	Danger-Associated Molecular Patterns
DCs	Dendritic Cells
DOX	Doxorubicin
DSPE-PEG2000	1,2-Distearoyl-sn-glycero-3-phosphoethanolamine-N-[methoxy(polyethylene glycol)-2000]
DSTAP	1,2-Dioleoyl-3-Trimethylammonium-Propane
DTX	Docetaxel
EPR	Enhanced Permeability and Retention
EGFR	Epidermal Growth Factor Receptor
EVs	Extracellular Vesicles
EMA	European Medicines Agency
FDA	U.S. Food and Drug Administration
FH	Factor H
GSH	Glutathione
GO	Graphene Oxide
GEM	Gemcitabine
GMP	Good Manufacturing Practice
GLP	Good Laboratory Practice
HCuSNPs	Hollow Copper Sulfide Nanoparticles
HER2	Human Epidermal Growth Factor Receptor 2
HO1	Heme Oxygenase 1
HPV	Human Papillomavirus
HSA	Human Serum Albumin
H_2_O_2_	Hydrogen peroxide
HA	Hyaluronic acid
HIF-1α	Hypoxia-inducible factor 1α
HepG2	hepatoblastoma cell line
ICD	Immunogenic Cell Death
IFN-γ	Interferon-gamma
IL-2	Interleukin-2
IND	Investigational New Drug
iNOS	Inducible Nitric Oxide Synthase
ICAM-1	Intercellular Adhesion Molecule 1
ICIs	Immune Checkpoint Inhibitors
IFNα	Interferon Alpha
IL-4R	Interleukin-4 Receptor
IFP	interstitial fluid pressure
IL-12	Interleukin 12
IL-10	Interleukin-10
IL-12@P1 NPs	IL-12-loaded poly (beta-amino ester) nanoparticles
IgE	Immunoglobulin E
LAG-3	Lymphocyte-Activation Gene 3
LFA-1	Lymphocyte Function-Associated Antigen 1
LPH	Liposome-Polycation-Hyaluronic Acid
LPHNPs	Lipid-Polymer Hybrid Nanoparticles
MDSCs	Myeloid-Derived Suppressor Cells
MHC	Major Histocompatibility Complex
MMPs	Matrix Metalloproteinases
MPa	Megapascal
MSNs	Mesoporous Silica Nanoparticles
MWCNTs	Multiwalled Carbon Nanotubes
mAbs	Monoclonal Antibodies
MBP	MnO_2_ Nanoparticles in PFOB Nanoemulsion
miRNA	MicroRNA
MRI	Magnetic Resonance Imaging
MUC4	Mucin 4
MDR	multidrug resistance
ML	Machine learning
MOFs	Metal–organic frameworks
MRPs	Multidrug resistance-associated proteins
MRX34	a liposome-encapsulated miR-34a mimic
NIR	Near-Infrared
NE	Nanoemulsion
NF-κB	Nuclear Factor Kappa-light-chain-enhancer of Activated B cells
NK	Natural Killer
NLCs	Nanostructured Lipid Carriers
NSCLC	Non-Small Cell Lung Cancer
NSCs	Neural Stem Cells
Ntn1	Netrin-1
NPs	nanoparticles
NO	Nitric oxide
NNM	nanoparticulate nanomedicines
ODN	Oligodeoxynucleotides
O2	Oxygen
PACA	Poly(Alkyl Cyanoacrylate)
PBCA	Poly(Butyl Cyanoacrylate)
PCL	Poly(ε-caprolactone)
PDT	Photodynamic Therapy
PEG	Polyethylene Glycol
PGA	Poly(γ-glutamic acid)
PLA	Polylactic Acid
PLGA	Poly(lactic-co-glycolic acid)
PTT	Photothermal Therapy
PD-1	Programmed Cell Death Protein 1
PD-L1	Programmed Death-Ligand 1
PEI	Polyethylenimine
PFOB	Perfluorooctyl Bromide
PLK1	Polo-like Kinase 1
pM330	Plasmid encoding Cas9 for Ntn1 targeting
poly(I:C)	Polyinosinic-Polycytidylic Acid
PTX	Paclitaxel
P-gp	P-glycoprotein
QDs	Quantum Dots
RGD	Arginine–Glycine–Aspartic Acid (Peptide)
ROS	Reactive Oxygen Species
R848	Resiquimod
RES	Reticuloendothelial System
RGD	Arginine-Glycine-Aspartic Acid
RICTOR	Rapamycin-Insensitive Companion of mTOR
RNS	Reactive nitrogen species
siRNA	Small Interfering RNA
SPIONs	Superparamagnetic Iron Oxide Nanoparticles
Sfn	Sorafenib
sgRNA	Single-Guide RNA
SIRP-α	Signal Regulatory Protein Alpha
SLNs	Solid Lipid Nanoparticles
SnMP	Tin Mesoporphyrin
STAT3	Signal Transducer and Activator of Transcription 3
STING	Stimulator of Interferon Genes
stPEI	Stearyl-Polyethylenimine
siRNA	Small Interfering Ribonucleic Acid
TAAs	Tumor-Associated Antigens
TCR	T-Cell Receptor
TERT	Telomerase Reverse Transcriptase
TILs	Tumor-Infiltrating Lymphocytes
TLR	Toll-Like Receptor
TME	Tumor Microenvironment
TNBC	Triple-Negative Breast Cancer
TAMs	Tumor-Associated Macrophages
TIGIT	T-cell immunoreceptor with Ig and ITIM domains
TIM-3	T-cell Immunoglobulin and Mucin-domain containing-3
Tregs	Regulatory T Cells
TriMix	A combination of mRNAs encoding CD70, CD40L, and constitutively active TLR4
TAFs	Tumor-associated fibroblasts
TAT	Trans-Activator of Transcription peptide
TNF-α	Tumor necrosis factor alpha
TAMs	Tumor-associate macrophages
TGF-β	Transforming growth factor beta
TMB	tumor mutational burden
ZP	Zeta Potential
